# Photoresponsive Adaptive Reconfiguration of Single‐Atom Interface With Intermittent Light and Soft Ionic Lattices

**DOI:** 10.1002/adma.202518557

**Published:** 2026-03-09

**Authors:** Li Yu, Jui‐Cheng Kao, Yuefeng Zhang, Chun Hong Mak, Yu‐Chieh Lo, Chun‐Wei Pao, Jyh‐Pin Chou, Zhenbin Wang, Ting‐Shan Chan, Hao Ming Chen, Hsien‐Yi Hsu

**Affiliations:** ^1^ School of Energy and Environment City University of Hong Kong Hong Kong China; ^2^ Department of Materials Science and Engineering National Yang Ming Chiao Tung University Hsinchu Taiwan; ^3^ Department of Materials Science and Engineering City University of Hong Kong Hong Kong China; ^4^ Research Center for Applied Sciences Academia Sinica Taipei Taiwan; ^5^ Graduate School of Advanced Technology National Taiwan University Taipei Taiwan; ^6^ National Synchrotron Radiation Research Center Hsinchu Taiwan; ^7^ Department of Chemistry & Center For Emerging Materials and Advanced Devices National Taiwan University Taipei Taiwan; ^8^ Center For Functional Photonics (CFP) City University of Hong Kong Hong Kong China; ^9^ Shenzhen Research Institute of City University of Hong Kong Shenzhen China

**Keywords:** dynamic reconfiguration, halide perovskite, intermittent light, single‐atom catalyst, soft ionic lattice

## Abstract

Achieving dynamic stability in single‐atom catalysts (SACs) is challenging, as it requires balancing strong metal‐support interactions with structural adaptability by incorporating flexibility into typically rigid SAC frameworks. Halide perovskites offer a unique platform for this purpose due to their soft ionic lattice and reversible dissolution–precipitation chemistry. We propose a concept for adaptively stabilizing SACs on halide perovskites through the integration of dynamic host chemistry, bandgap engineering, and light‐regulated metal speciation. A light‐induced bandgap funneling effect guides photogenerated carriers to deposit atomic platinum under illumination, while the dynamic interface prevents premature clustering during dark periods by refreshing the catalytic surface. The ionic, electronic, and atomic structural synergy enables a programmable intermittent illumination strategy, which drives continuous renewal of the interfacial atomic configuration and sustains high activity in hydrogen halide splitting and hydrogen production over multiple cycles. This work provides fundamental insights into adaptive catalytic interfaces and suggests new pathways for smart photocatalyst engineering via dynamic material‐light interplay.

## Graphical abstract

We present a photoresponsive platform that integrates SACs with halide perovskite supports, turning perovskite ion mobility, typically a stability concern, into a functional mechanism for light‐regulated self‐repair and reversible atomic reconfiguration. Adaptive metal‐support interactions are established through bandgap‐funnel engineering and the soft ionic lattice under dynamic illumination.

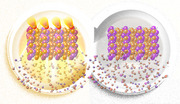

## 1 Introduction

Single‐atom catalysts (SACs) exhibit high catalytic efficiency and selectivity due to their atomically dispersed active sites, which maximize the utilization of metal atoms [1, 2, 3, 4, 5, 6]. However, these atomic configurations are susceptible to irreversible structural degradation during catalysis, leading to a significant decline in their long‐term performance [7, 8, 9, 10]. Mechanistic investigations indicate that the catalytic functionality and stability of single‐atom sites are directly influenced by dynamic changes in their local coordination environment, including coordination geometry and electronic states [11, 12, 13]. The instability originates primarily from thermodynamic constraints: their inherently high surface energy drives aggregation into nanoparticles [14, 15], while weak metal‐support interactions make them vulnerable to dissolution under reactive conditions [16]. The detrimental effects of the structural instabilities become particularly pronounced under harsh conditions typical of many catalytic processes, such as extreme pH levels and reactive environments, where uncontrolled atomic‐scale rearrangements result in unpredictable variations in catalytic activity [17, 18].

In contrast to the rigid nature of typical covalent bonds in SACs, dynamic interactions offer adaptability to changing conditions [19, 20]. Reversible or dynamic covalent chemistry has long been a well‐established concept in polymer science [21, 22, 23]. The inherent ability of dynamic bonds to reversibly dissociate and reassociate confers upon polymeric systems considerable structural flexibility, allowing them to adjust their architecture or composition in response to external stimuli [24]. Applying this principle at the atomic scale in SACs may lead to new techniques, such as support materials engineered to immobilize and redistribute single atoms, or tailored reaction settings that enable the redispersion of aggregated metallic species. However, achieving dynamic bonding in SACs is difficult due to the intricate balance required between strong metal‐support interactions and the adaptability necessary for such bonding [25, 26]. The complexity stems from the inherent contradiction of integrating flexibility within otherwise inflexible frameworks.

Halide‐based perovskites are recognized for their soft ionic lattice structures and dynamic equilibrium in saturated solutions [27, 28, 29, 30, 31, 32]. The flexibility of the ionic lattice allows it to accommodate and stabilize single atoms of varying sizes and charges. A dynamic ionic environment facilitates the reversible incorporation and liberation of ions, affording precise control over the composition and distribution of single atoms within perovskites. While earlier research has elucidated the dynamic equilibrium of halide perovskites in acidic media [32] and their capacity to host SACs [33], this work merges these concepts into a unified, light‐adaptive system. We demonstrate how the integration of dynamic host chemistry, a compositional bandgap funnel, and photoregulated single‐atom behavior enables sustained catalytic function through continuous interface regeneration.

## 2 Results and Discussion

### 2.1 Bandgap‐Funnel Structure for Pt Single‐Atom Immobilization

**FIGURE 1:**
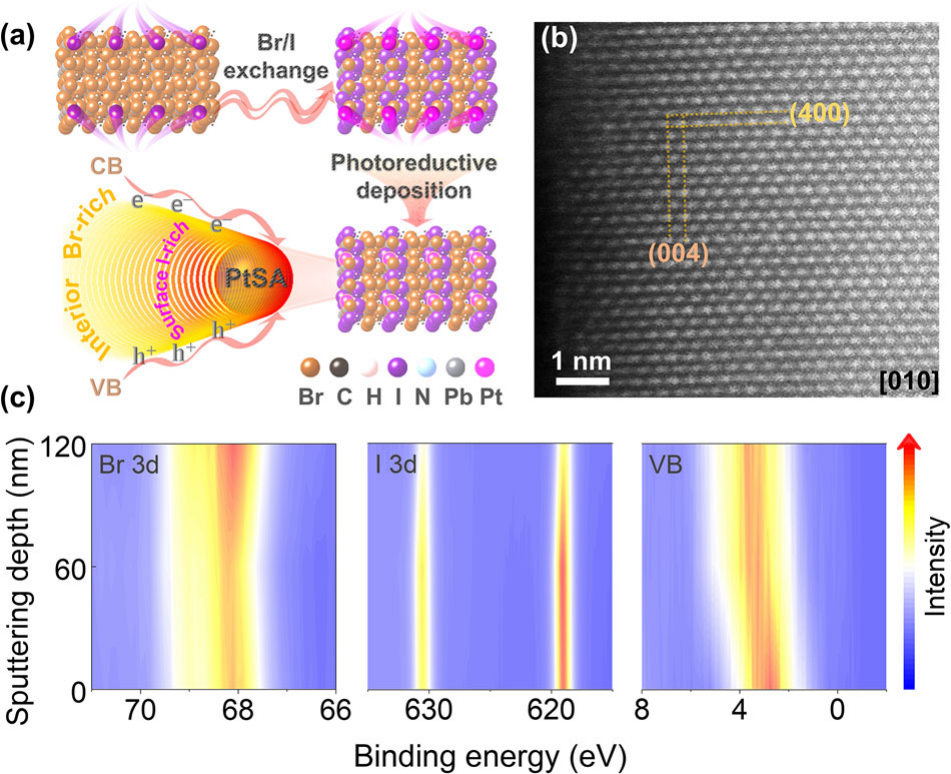
Bandgap‐funnel structure for Pt single‐atom immobilization. (a) Schematic of bandgap‐funnel MPBI synthesis via halide exchange from MPB and subsequent single‐atom Pt anchoring. The gradient halide composition optimizes carrier transport from the conduction band (CB) and valence band (VB) to stabilize Pt sites. (b) Atomic‐resolution STEM image of MPBI along the [010] zone axis. (c) Depth profile analysis of background‐corrected Br 3d, I 3d, and VB XPS spectra for MPBI, visualized through color‐mapped intensity contours.

**FIGURE 2:**
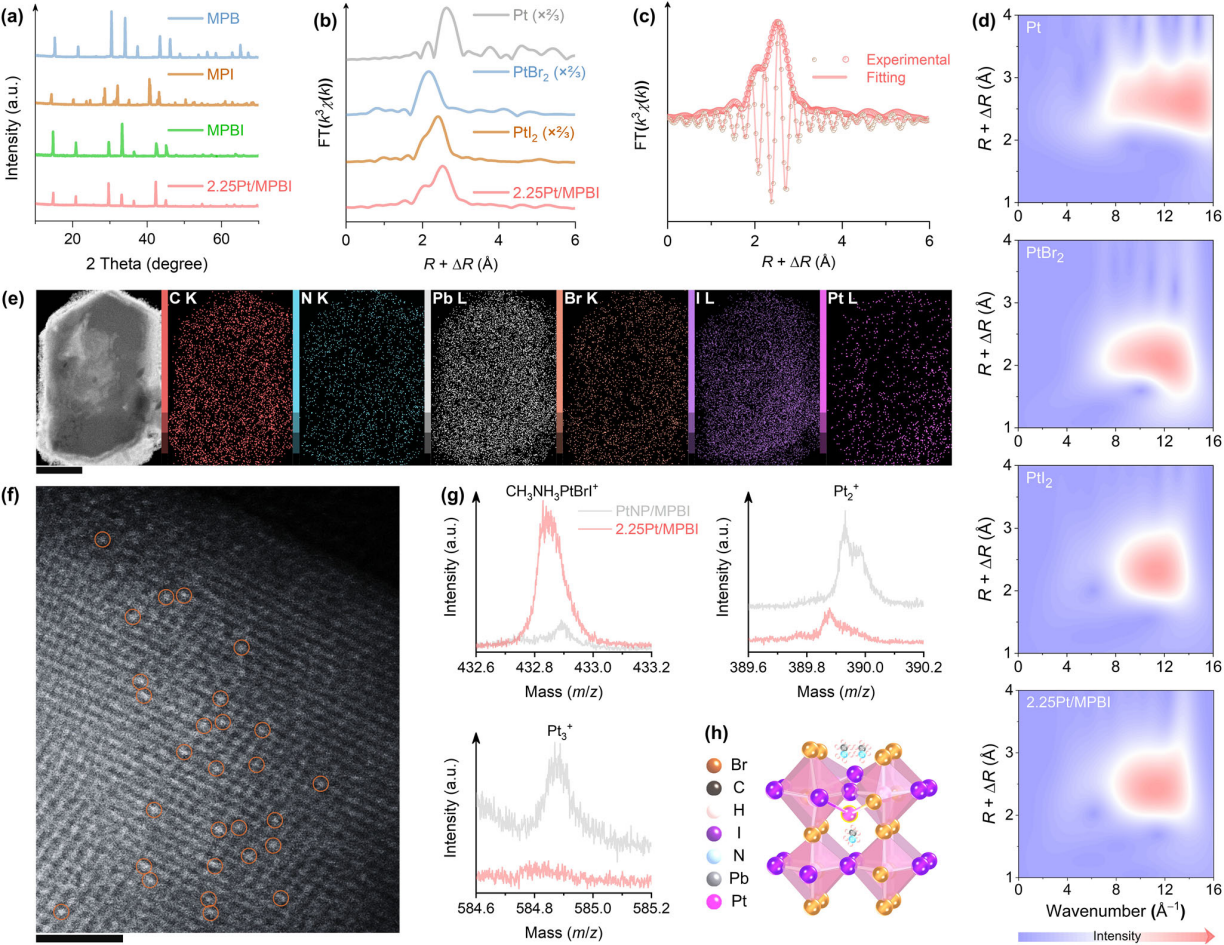
Multiscale structure resolving: From bulk phase to atomic configuration and surface chemistry. (a) XRD patterns. (b) Fourier transform (FT) *k* ^3^‐weighted χ(*k*)‐function of the Pt *L*_3_‐edge EXAFS spectra. (c) Theoretical magnitude and real part of the FT EXAFS profiles compared to the experimental spectra. (d) Experimental 2D WT EXAFS plots for *χ*(*k*) data. (e) EDXS elemental mapping of 2.25Pt/MPBI (scale bar: 1 µm). (f) Atomically dispersed Pt sites in 2.25Pt/MPBI visualized by aberration‐corrected HAADF‐STEM, with brighter spots circled (scale bar: 2 nm). (g) ToF‐SIMS fragment ion distributions. (h) Proposed structural model of Pt coordination in MPBI.

We develop a bandgap‐funnel structure in MAPb(Br_x_I_1−x_)_3_ (MPBI; MA = methylammonium cation) to stabilize Pt single atoms (Pt‐SAs) via a light‐assisted halide exchange strategy. The synthesis involves treating MAPbBr_3_ (MPB) particles in an aqueous mixture of hydrobromic acid (HBr) and hydroiodic acid (HI), followed by light‐driven halide redistribution to form MPBI with a graded composition (Figure 1a). Atomic‐resolution spherical aberration‐corrected scanning transmission electron microscopy (AC‐STEM) reveals well‐defined lattice structures on the MPBI surface (Figure 1b). To minimize electron beam‐induced damage, imaging parameters are optimized in adjacent regions prior to data acquisition [34]. The orthogonal lattice fringes with 2.20 and 3.18 Å spacings, which match the (400) and (004) planes of MAPbI_3_ (MPI), provide evidence of bromide‐to‐iodide substitution at the surface (Figure 1b; Figure S1). X‐ray photoelectron spectroscopy (XPS) depth profiling reveals a progressive increase in surface iodide content accompanied by a decrease in bromide from the bulk to the surface (Figure 1c). In MPBI, the bulk cubic structure is retained, but its peaks shift slightly to lower angles relative to MPB due to the partial replacement of bromide with larger iodide ions (Figure 2a). The compositional gradient induces an intermediate bandgap of 1.96 eV for MPBI, positioned between MPB and MPI (Figure S2). In addition, depth‐dependent XPS valence band measurements unveil a gradual positive shift in the valence band maximum (VBM) with increasing depth, directly visualizing the bandgap gradient critical for charge carrier funneling.

The bandgap funneling effect is anticipated to facilitate Pt‐SA stabilization on the MPBI surface (Pt‐SA/MPBI), driven by directional charge carrier migration from bromide‐rich interiors to iodide‐rich surfaces through aligned energy levels. To probe this mechanism, Pt‐SA/MPBI samples with controlled Pt loadings (denoted as *ω*Pt/MPBI, where *ω* represents the weight percentage of Pt added relative to the perovskite matrix) are synthesized. X‐ray diffraction (XRD) analysis reveals no peak shifts in 2.25Pt/MPBI compared to pristine MPBI, ruling out crystalline Pt formation. Progressive attenuation of XRD peak intensities from MPB to MPBI and further to 2.25Pt/MPBI correlates with increasing iodide substitution. During the photochemical synthesis of 2.25Pt/MPBI from MPBI, the surface iodine content increases while bromine decreases (Figure S3). 2.25Pt/MPBI retains the primary optical absorption characteristics of MPBI but exhibits a slight red shift (Figure S2). The optical behavior confirms the preservation of the perovskite framework during Pt deposition, accompanied by an enhanced bromine‐iodine compositional gradient at the surface due to iodine enrichment. The intensified compositional gradient enhances the bandgap funneling effect to promote Pt‐SA formation. In MPBI, the spatially varying potential landscape (Figure 1a) drives photogenerated electrons to accumulate in the iodide‐rich surface region and generate localized electron‐rich environments, which in turn stabilize Pt ions through charge compensation. Conversely, homogeneous halide distributions in MPB or MPI fail to generate such potential gradients, resulting in insufficient driving force for Pt loading on 2.25Pt/MPB and 2.25Pt/MPI (Figure S4). The observed depth‐dependent attenuation of Pt 4f XPS signals demonstrates preferential Pt anchoring at the surface (Figure S5). In infrared spectral analysis (Figure S6), 2.25Pt/MPBI exhibits surface vibrational modes closely resembling those of pristine MPBI, indicating minimal alterations to surface chemical functionality upon Pt incorporation. The lack of chloride incorporation from the Pt precursor is attributed to its low concentration relative to the large excess of bromide/iodide and to its comparatively slow exchange kinetics, as supported by XPS analysis (Figure S7).

Synchrotron‐radiation‐based Pt *L*_3_‐edge X‐ray absorption near‐edge structure (XANES) analysis of 2.25Pt /MPBI (Figure S8) reveals a prominent white‐line peak, characteristic of electronic transition from the filled Pt 2*p*_3/2_ core level to the unoccupied 5*d* orbitals [35]. The white‐line intensity of 2.25Pt/MPBI differs markedly from metallic Pt foil and PtO_2_, instead exhibiting a close similarity to PtBr_2_ and PtI_2_ standards. Differential XANES (ΔXANES) quantification further identifies *d*‐band hole concentrations comparable to those of PtBr_2_ and PtI_2_, as determined by integrating the white‐line peak area (Figure S9). Compared to metallic Pt, 2.25Pt/MPBI exhibits significantly enhanced *d*‐band hole density and a quantifiable increase in the valence state of Pt. The electronic restructuring manifests as a distinct deviation from the typical electronic configuration of metallic Pt. During the photoreduction synthesis of Pt‐SAs, the ligand‐to‐metal charge transfer (LMCT) facilitates electron donation from halide perovskite matrices to Pt cations. This charge redistribution establishes robust covalent Pt‐halide bonds that stabilize Pt atoms within the hybrid perovskite framework. Fourier transform (FT) analysis of extended X‐ray absorption fine structure (EXAFS, Figure 2b) resolves two distinct coordination features: a dominant peak at ∼2.5 Å and a shoulder at ∼2.2 Å (phase uncorrected). The shorter distance corresponds to Pt─Br bonding, while the 2.5 Å peak lies between Pt–I and Pt–Pt reference paths. Given the XANES similarity to PtBr_2_/PtI_2_ rather than metallic Pt, we modeled the first coordination shell with Pt–Br and Pt–I paths. Least‐squares fitting of the *R*‐space EXAFS magnitude and real components gives coordination numbers of 0.8 for Pt–Br and 1.3 for Pt–I (Figure 2c), demonstrating Pt single‐atom stabilization via mixed halide coordination.

While FT‐EXAFS has difficulty in distinguishing between Pt–Pt and Pt–I coordination paths due to their similar radial distances, wavelet transform (WT) analysis enables clear differentiation by resolving atomic‐specific backscattering signatures in *k*‐space. The backscattering amplitude depends strongly on atomic number—heavier elements (e.g., Pt) dominate at higher wavenumbers compared to lighter ones [36]. WT‐EXAFS analysis of 2.25Pt/MPBI reveals distinct lobe centers at ∼12.0 Å^−1^, closely matching the Pt–I/Pt–Br coordination range observed in PtI_2_ and PtBr_2_ standards, whereas metallic Pt−Pt bonds appear at ∼14.0 Å^−1^ (Figure 2d). This wavenumber localization, combined with contour map profiles closely resembling PtI_2_, rules out Pt–Pt metallic bonding. As expected, 2.25Pt/MPBI is characterized by a homogenous elemental distribution in energy‐dispersive X‐ray spectroscopy (EDXS) mapping (Figure 2e) and an atomically dispersed state of Pt, as resolved by aberration‐corrected high‐angle annular dark‐field STEM (HAADF‐STEM, Figure 2f). To complement the structural insights obtained from XANES, FT‐EXAFS, and WT‐EXAFS analyses, we employ time‐of‐flight secondary ion mass spectrometry (ToF‐SIMS) to directly probe the surface coordination chemistry of Pt species. ToF‐SIMS analyses of Pt‐SA/MPBI (2.25Pt/MPBI) and Pt nanoparticle‐MPBI mixture (PtNP/MPBI) reveal distinct differences in their surface chemical signatures (Figure 2g). Both systems exhibit comparable Pt^+^ fragment signals (Figure S10), but 2.25Pt/MPBI shows significantly higher intensities for CH_3_NH_3_PtBrI^+^ fragments and substantially lower signals for Pt_2_^+^ species, with Pt_3_^+^ fragments nearly undetectable. In contrast, PtNP/MPBI displays pronounced Pt_2_^+^ and Pt_3_^+^ signals alongside minimal CH_3_NH_3_PtBrI^+^ fragments. Such a significant difference highlights that atomic Pt in 2.25Pt/MPBI predominantly coordinates with Br and I ions within the perovskite lattice, while Pt nanoparticles in PtNP/MPBI lack such coordination. These results conclusively validate the atomic‐level coordination structure of Pt in 2.25Pt/MPBI, as illustrated in the proposed model (Figure 2h).

### 2.2 Pt SA Immobilization Triggering Fast Carrier Dynamics

**FIGURE 3:**
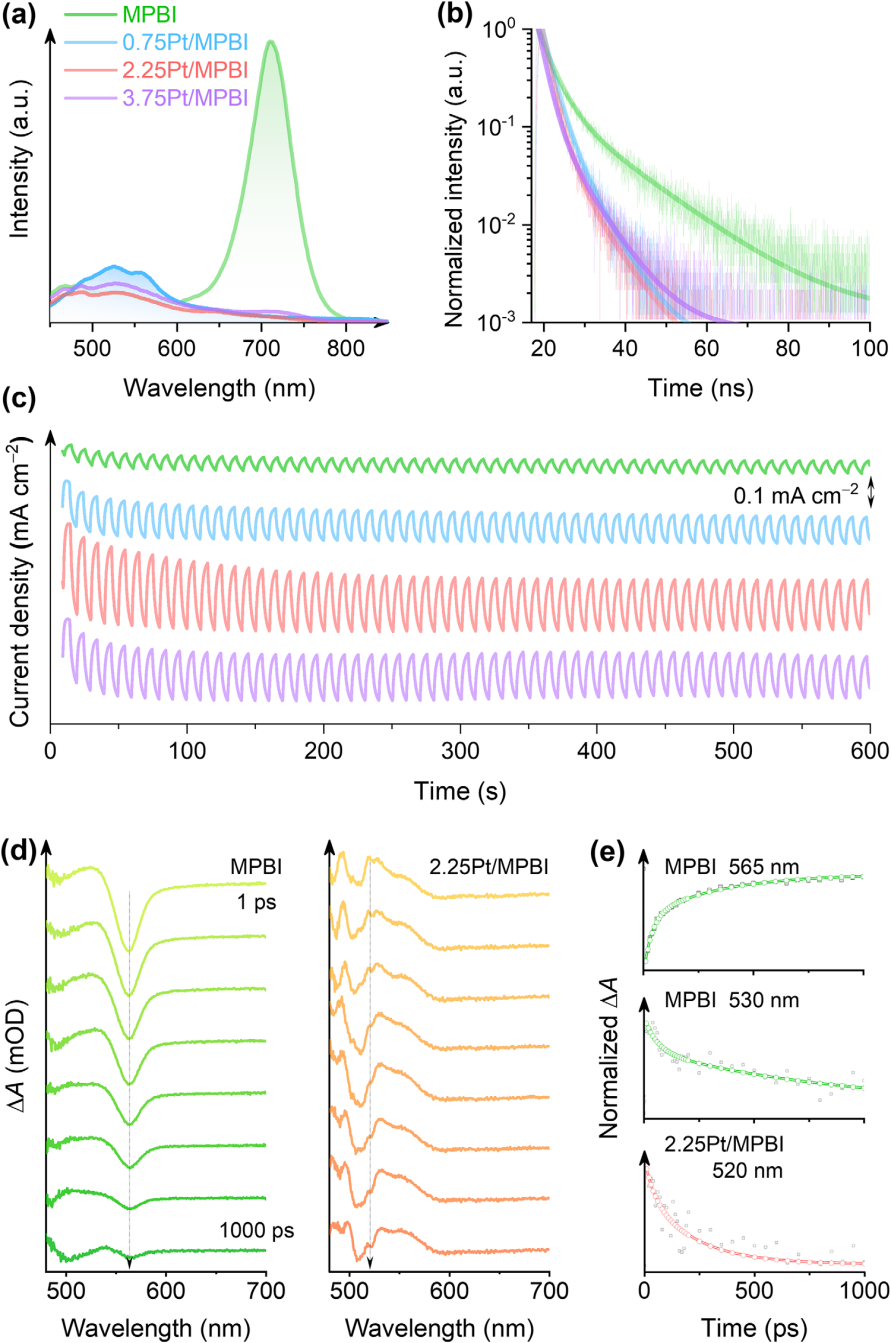
Pt single‐atom immobilization triggering fast carrier dynamics. (a) Static PL spectra. (b) Time‐resolved PL spectra. (c) Chopped photocurrent profiles. (d) Time‐resolved TAS spectra of MPBI and 2.25Pt/MPBI at selected delay times. (e) Time‐resolved kinetics decay at selected wavelengths with exponential‐function fits.

The charge carrier dynamics within Pt‐SA/MPBI are probed through steady‐state photoluminescence (PL) spectroscopy. The analysis reveals two distinct spectral signatures: a broad asymmetric emission band at 566 nm corresponding to the MPB phase, accompanied by a resolved emission peak at 710 nm characteristic of the MPI component (Figure 3a). Compared to the PL emission near 566 nm, even minimal Pt deposition significantly reduces the fluorescence intensity at 710 nm. Within the framework of static fluorescence quenching, Pt SAs are in proximity to the luminescent centers of the perovskite and interact with adjacent iodine atoms through orbital coupling, causing the observed quenching near 710 nm [37]. The PL decay profile, monitored at 710 nm, indicates an average PL lifetime of 5.4 ns for MPBI, whereas Pt‐SA/MPBIs display significantly shorter lifetimes, spanning from 2.5 to 2.9 ns (Figure 3b; Table S1). The decrease in both PL intensity and lifetime is attributed to the disruption of electron‐hole pair recombination on Pt‐SA/MPBI, wherein surface Pt atoms act as charge carrier traps [33, 38, 39].

Electrochemical impedance spectroscopy (Figure S11) analysis identifies three distinct semicircular features in the Nyquist plots, corresponding sequentially to (1) resistance and associated capacitance at the electrode/electrolyte interphase layers, (2) electronic conduction within materials, and (3) Faradaic charge transfer processes. The charge conduction between the electrolyte and electrode interfaces exhibits stable behavior across all samples. Incorporation of Pt SAs significantly enhances bulk electronic conduction, suggesting the formation of an interconnected electron/ion transport network within the electrode matrix. Specifically, 2.25Pt/MPBI demonstrates a marked improvement in Faradaic charge‐transfer kinetics, as evidenced by its substantially reduced charge transfer resistance (∼1609 Ω cm^2^, Table S2). Cyclic voltammetry profiles reveal that the electron transfer process is predominantly diffusion‐controlled (Figures S12–S15). The presence of Pt SAs remarkably enhances the interfacial kinetic processes driven by either oxidation or reduction potentials. In particular, the kinetic parameters associated with 2.25Pt/MPBI are consistently higher by factors ranging from several‐fold to an order of magnitude compared to the MPBI system (Table S3). A robust photocurrent response is observed for the photoelectrode fabricated by coating 2.25Pt/MPBI powder onto fluorine‐doped tin oxide (FTO) conductive glass, with stable chopped photocurrent profiles across multiple illumination on‐off cycles (Figure 3c). At an applied bias of −0.5 V vs. Ag/Ag^+^, the photoelectrode exhibits a sustained photocurrent density of 0.17 mA cm^−2^, representing about 4‐fold improvement in photoelectrochemical performance relative to the MPBI|FTO control (0.042 mA cm^−2^) after prolonged cycling (600 s).

In addition to the photoelectrochemical and physical characterization of carrier migration mechanisms, transient absorption spectroscopy (TAS) provides direct insight into the ultrafast excited‐state dynamics of MPBI and Pt‐SA/MPBI systems. A pronounced negative absorption band appears near 565 nm in pristine MPBI, corresponding to stimulated emission from the excited state. This feature shows biphasic recovery kinetics with time constants of 36.8 and 334 ps (Figure 3d,e), indicative of sequential relaxation pathways to the ground state. An approximately baseline‐symmetric intensity correlation observed between the spectral profile and the MPB‐phase steady‐state emission identifies radiative recombination as the underlying mechanism. In 2.25Pt/MPBI, the stimulated emission signature disappears, indicating that atomically dispersed Pt sites reconfigure the excited‐state relaxation channels of the perovskite host. Such alteration arises from the modified electronic coupling between the perovskite matrix and Pt centers via Pt–Br coordination, which redirects carriers toward non‐radiative energy transfer pathways. Both systems further exhibit positive absorption signals from photoinduced absorption (PIA) processes. Pristine MPBI shows a PIA band centered near 530 nm with biphasic decay kinetics (59.6 and 871 ps). In comparison, 2.25Pt/MPBI displays a blue‐shifted PIA feature at approximately 520 nm with faster dynamics, characterized by reduced time constants of 43.2 and 218 ps. The biphasic relaxation behavior corresponds to electron trapping at different energetic depths, with the faster and slower components attributed to shallow trap states and deeper trap‐mediated recombination, respectively [40]. The shorter PIA lifetimes in 2.25Pt/MPBI indicate more efficient electron trapping and charge separation. By suppressing radiative electron‐hole recombination via Pt‐induced charge trapping states, these kinetic modifications offer the potential to enhance proton reduction efficiency for hydrogen evolution.

### 2.3 Efficient and Sustained Photocatalytic Hydrogen Evolution Reactions (PHER)

**FIGURE 4:**
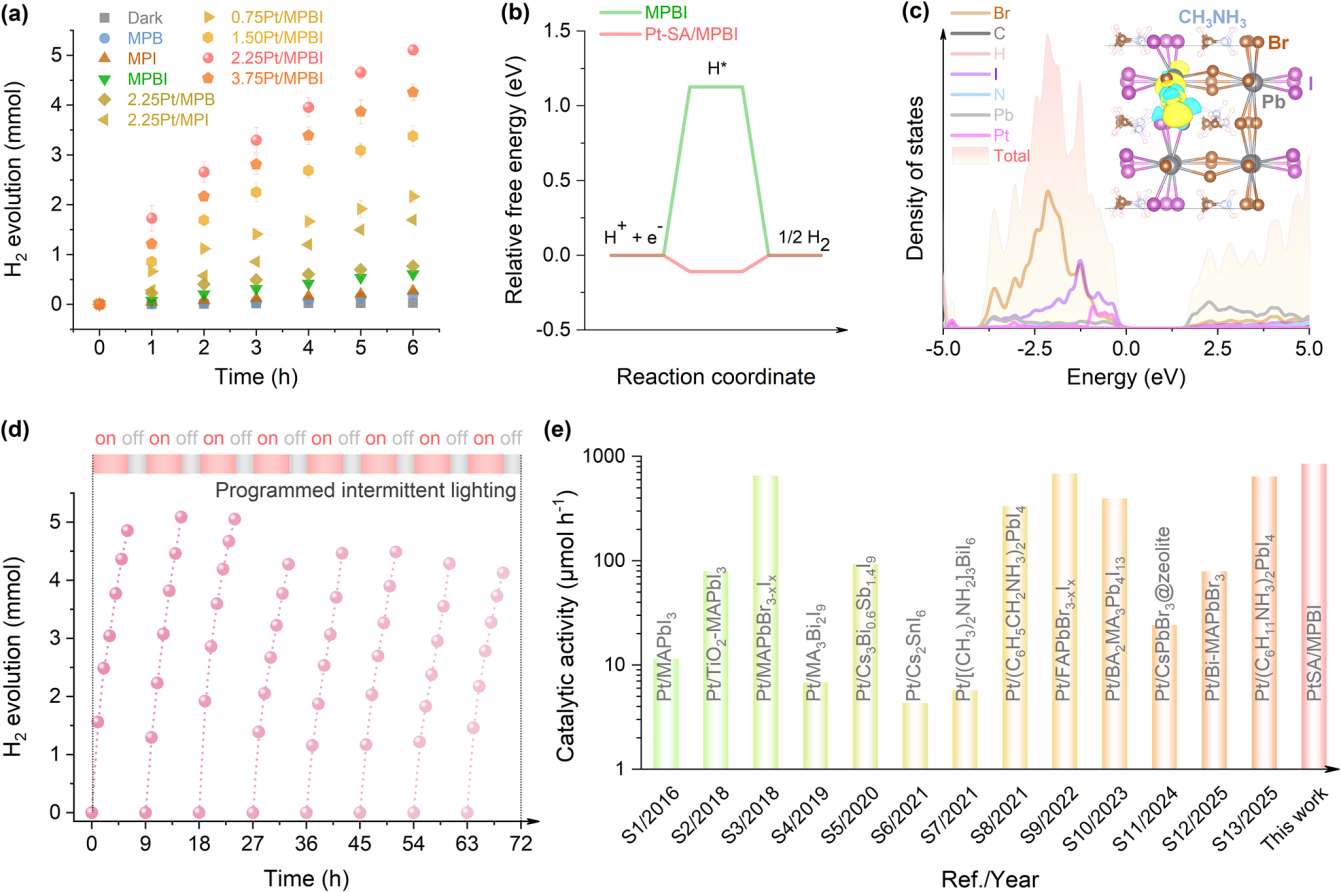
Synergistic experimental‐theoretical insights into high‐efficiency hydrogen halide splitting for sustainable hydrogen production. (a) Time‐dependent PHER under visible light. (b) Free energy profiles for HER. (c) Density of states (DOS) and charge density difference analyses. Cyan and yellow regions indicate electron depletion and gain, set at an isosurface of 0.001 e/Å^3^. (d) Long‐term durability of 2.25Pt/MPBI under programmed light‐dark cycling (6 h ON/3 h OFF) during 72‐hour continuous operation. (e) Comparative performance benchmarking of visible‐light‐driven hydrogen evolution in Pt‐modified perovskite composites against published systems (ref. S1–S13).

With Pt‐SA incorporation, the observed improvement in carrier dynamics directly translates to enhanced PHER performance, as measured under visible light irradiation (Figure S16). MPI exhibits limited PHER activity, achieving an average rate of 44.1 µmol h^−1^ over six hours (Figure 4a). This performance is moderately enhanced to 102 µmol h^−1^ for the MPBI bandgap‐engineered structure, attributed to improved photogenerated charge carrier migration. Increasing the Pt loading from 0.75 to 2.25 wt.% boosts photocatalytic activity, reaching an average rate of 851 µmol h^−1^ over six hours and a high apparent quantum yield (AQY) of 15.5% at 450 ± 16 nm (Figure S17). In addition, 2.25Pt/MPBI exhibits a notable solar HI splitting efficiency of approximately 1.0%, indicating the potential for efficient solar‐to‐hydrogen conversion. However, subsequent increases in Pt loading diminish the photocatalytic activity in 3.75Pt/MPBI despite its predominant structural similarity to 2.25Pt/MPBI (Figures S10, S18, and S19). The activity reduction correlates with progressively stronger visible light absorption in the precursor solution as Pt loading rises from 2.25 to 3.75 wt.% (Figure S20a). The dissolved Pt complexes attenuate incident photon flux through competitive light absorption, leading to unwanted energy loss that reduces effective light intensity at the catalyst surface. A compensatory effect of increased light intensity further indicates that differential light absorption is a key mechanistic factor behind the observed performance trend (Figure S20b).

Evaluation of hydrogen adsorption energetics across different sites shows that Pt sites in Pt‐SA/MPBI exhibit the most thermodynamically favorable adsorption configuration, whereas MPBI lacks effective sites for efficient hydrogen adsorption (Figure S21). The formation of hydrogen intermediates (H^*^) on pristine MPBI is an endergonic process with a substantial Gibbs free energy change (ΔG) of 1.13 eV, marking it as the rate‐determining step (RDS) for HER (Figure 4b). Electronic structure analysis indicates the valence band top of MPBI primarily consists of halide *p* orbitals mixed with lead states, while the conduction band features dominant contributions from lead *p* orbitals partially hybridized with halide states (Figure 4c). Hybridization between Pt and halogen (I/Br) orbitals generates new electronic states near VBM that simultaneously enhance charge carrier trapping and suppress detrimental electron‐hole recombination. The introduction of single‐atom Pt consequently promotes HER activity by enabling thermodynamically favorable proton‐electron transfer kinetics. During this process, one H^*^ gains 0.68 electrons from the substrate toward an efficient reaction pathway. The RDS for Pt‐SA/MPBI entails the dimerization of H^*^ to yield H_2_ in a much‐decreased ΔG of 0.11 eV, indicative of its superior HER performance.

The photocatalyst's stability was assessed through repeated photocatalytic cycles, each consisting of 6 h of exposure to visible light followed by 3 h of darkness. PHER is most effective during the initial hour, attaining a rate of 1.6 mmol h^−1^ (Figure 4d). The superior photocatalytic performance stems from the synergistic integration of a bandgap‐funnel architecture with atomically dispersed Pt coordination centers, which collectively enhance charge carrier separation while accelerating interfacial reaction kinetics. The unique structural‐electronic configuration enables the system to surpass conventional Pt‐modified perovskite composites in overall activity, as demonstrated through comparative evaluations (Figure 4e). While extended light exposure modestly reduces catalytic activity, this effect is reversible after each cycle. The system preserves its high activity over eight consecutive reaction cycles, with a consistent PHER rate of 1.5 mmol h^−1^ in the first hour of a subsequent new cycle of visible‐light photocatalysis.

### 2.4 Self‐Adaptive Behavior of Single‐Atom Pt

**FIGURE 5:**
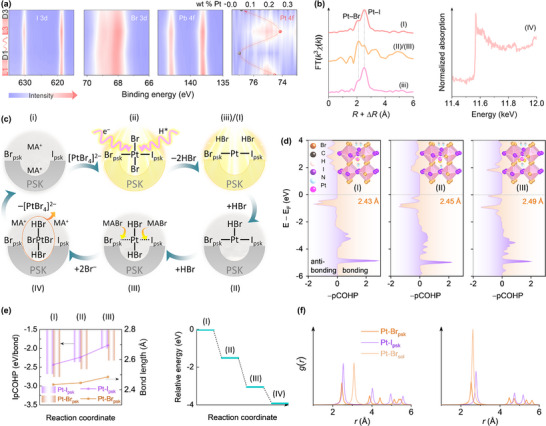
Dynamic coordination chemistry and self‐regenerative behavior of single‐atom Pt. (a) Contour mapping of high‐resolution background‐corrected XPS profiles under programmed light‐dark cycling. The inset arrow illustrates the corresponding variation in surface Pt content measured by ICP‐OES. (b) XAFS‐derived coordination evolution of Pt single atoms during alternating light and dark phases. (c) Schematic illustration of the proposed reversible coordination changes at the perovskite interface under cyclic illumination. (d–f) Theoretical analysis of Pt dissolution dynamics at the perovskite–acid interface. (d) pCOHP analysis for the Pt–Br_psk_ bonding pair under different HBr adsorption scenarios, with corresponding structural configurations and bond lengths shown in the accompanying figures. (e) IpCOHP and bond lengths for Pt−Br_psk_ and Pt−I_psk_ bonding pairs, alongside the free energy profile for sequential HBr adsorption and Pt dissolution. (f) Pt‐ligand RDFs showing the transition from the initial to the final evolved configuration within 1 ps.

The inherent benefits of halide perovskite, such as its reconfigurable structural framework and soft ionic lattice, motivate our exploration into the stability origin of Pt‐SA/MPBIs' photocatalytic activity. Subsequent experiments were designed to examine the structural transformations of Pt at various stages: following initial illumination (2.25Pt/MPBI), after the first light‐dark cycle (2.25Pt/MPBI_D1), the third illumination (2.25Pt/MPBI_L3), and the third light‐dark cycle (2.25Pt/MPBI_D3). Our XPS analysis across the Br 3d, I 3d, and Pb 4f core levels reveals no significant changes in peak position and intensity (Figure 5a), and VBM maintains a consistent position at approximately 1.1 eV below the Fermi level (E_F_) (Figure S22). However, 2.25Pt/MPBI_D1 and 2.25Pt/MPBI_D3 exhibit comparable Pt 4f peak intensities, notably lower than those observed for 2.25Pt/MPBI_L1 and 2.25Pt/MPBI_L3. The observation suggests light exposure promotes Pt deposition, while Pt atom migration into the acidic medium occurs during dark periods, analogous to the behavior of the Cu/(CuI_2_)^−^ couple in aqueous hydroiodic acid [41]. To verify the proposed Pt dissolution and reassociation dynamics, we tracked the Pt content in both the solid and solution phases during light–dark cycling using inductively coupled plasma optical emission spectroscopy (ICP‐OES). The total Pt mass across the two phases shows minimal change throughout the cycle (Figure S23), confirming a reversible redistribution between the surface and the solution rather than irreversible loss or accumulation. These measurements reveal cyclic Pt dissolution in the dark and redeposition under illumination, a trend consistent with the Pt content variations observed by XPS between light and dark states (Figure 5a). The efficient regeneration of Pt, which refers to its ability to re‐enter the catalytic cycle, is thus maintained under the dynamic conditions of our system. Together with the quantified rates of light‑driven redeposition and dark‑state dissolution, these results support an intermittent illumination strategy that effectively sustains the regenerative single‑atom state.

Further investigation into the dynamic behavior of Pt SAs in the MPBI matrix under alternating light‐dark cycles within HBr‐dominated media reveals a reversible structural evolution process (Figure 5b,c). Initial EXAFS analysis confirms the atomic dispersion of Pt species in the pristine catalyst, characterized by dominant Pt−Br/I coordination shells. After maintaining the system under dark conditions for 1 h, we detect an enhancement in Pt−Br coordination intensity along with intensified scattering signals beyond 3.0 Å. These changes reveal progressive chemical interactions between Pt species and concentrated HBr during dark exposure, primarily through the formation of soluble Pt−Br complexes. The extended‐range disorder emerges from coordination environments beyond the first shell, reflecting increased positional disorder of Pt moieties within the matrix. A complete light‐dark cycle ultimately leads to substantial Pt dissolution, as evidenced by the ineffective EXAFS spectra that preclude precise coordination environment determination. Although the spectral quality limits detailed structural analysis at this stage, the signal degradation itself provides critical indirect evidence supporting the light‐modulated dissolution‐redeposition mechanism. Subsequent re‐illumination regenerates the single‐atom Pt configuration, with the restored coordination environment closely resembling the initial atomic dispersion state. This cyclical regeneration demonstrates the dynamic equilibrium between light‐driven Pt redeposition and dark‐phase dissolution, mediated through bromide‐assisted complexation. The minor variations in XRD peak profiles observed after extended cycling originate from reversible microstructural rearrangements within the dynamic perovskite lattice. These changes do not affect the catalytic function and can be fully remediated through the system's inherent recrystallization capability (Figure S24).

Given the bromide‐rich environment, the introduced Pt species predominantly exist as [PtBr_6_]^2−^ complexes. Under light illumination, these complexes undergo stepwise photodriven reduction. Photogenerated electrons first reduce [PtBr_6_]^2−^ to [PtBr_4_]^2−^. Once [PtBr_4_]^2−^ adsorbs onto the MPBI surface, it releases two bromide ligands, forming a key intermediate in which a single [PtBr_2_] unit becomes anchored via both Pt─Br and Pt─I bonds to the perovskite surface (Figure 5c). Concurrently, in the highly acidic medium, protons are reduced at the Pt sites under illumination to form atomic hydrogen. The injection of electrons into the Pt center significantly weakens the Pt─Br bonds. Atomic hydrogen can then react with the bromide ligands to form HBr, which desorbs from the surface. This step corresponds to the characteristic photoreductive deposition of metal atoms, driving the stabilization of Pt single atoms through Pt–Br and Pt–I coordination with the perovskite lattice. It is noteworthy that Pt–Br and Pt–I coordination alone would be inherently unstable in a bromide‐rich solution due to possible ligand exchange or HBr‐induced cleavage. However, under continuous illumination, the Pt site undergoes periodic electron injection, which persistently removes excess adsorbed bromide ligands via the hydrogen‐assisted desorption pathway. The light‐mediated cycle suppresses over‐coordination and bromide‐induced dissolution, stabilizing the Pt single‐atom configuration against halide exchange or acidic etching.

In contrast, the absence of this hydrogen‐assisted desorption pathway under dark conditions allows over‐coordination of Pt sites by bromide, ultimately causing dissolution of the surface Pt species (Figure 5d,e). The adsorption of HBr molecules at Pt sites is energetically favorable and barrier‐free. Projected crystal orbital Hamilton population (pCOHP) analysis further elucidates the nature of interactions between Pt and Br atoms within the perovskite structure (Pt−Br_psk_) [16, 42, 43]. The positive and negative regions of the −COHP function correspond to bonding and antibonding interactions, respectively, while values approaching zero signify nonbonding interactions. Upon adsorption of HBr, there is a noticeable decrease in the occupancy of the Pt−Br_psk_ bonding state within the VB region, suggesting a reduction in the bond strength of Pt−Br_psk_. In addition, the antibonding peak associated with Pt−Br_psk_ shifts closer to E_F_ after adsorption. The proximity to E_F_ results in an increase in the system's total energy and, consequently, a structurally unstable configuration [44].

As HBr adsorption increases, the absolute integrated pCOHP (|IpCOHP|) for Pt−Br_psk_ decreases while the bond lengthens, indicating progressive bond weakening. This weakening is corroborated by numerical data showing elongated bond lengths and reduced |IpCOHP| values for Pt−I_psk_ (Figure S25), which also leads to the leaching of Pt SAs. When the Pt−Br_psk_ and Pt−I_psk_ bonds undergo sufficient weakening, they can be attacked by additional bromide species. Ion exchange with MABr as the bromide source may contribute to the final dissolution step. In this scenario, the cationic MA^+^ species primarily serves to maintain charge balance within the perovskite structure during the removal of bromide ions, while the released bromide ions actively participate in the coordination sphere of the Pt center. The exchange process effectively lowers the kinetic barrier for Pt detachment and contributes to the formation of the final [PtBr_4_]^2−^ complex in solution. Our preliminary energetic assessments confirm that this pathway remains thermodynamically feasible, consistent with the observed experimental dissolution behavior. However, the predominant driving force for the dissolution mechanism originates from the strong coordinative interaction between Pt and bromide, rather than from direct involvement of the organic cation. The process frees up the perovskite surface sites for a new cycle of Pt photodeposition, which prevents the premature formation of Pt clusters. Such clusters could otherwise arise from interactions between newly formed Pt and previously deposited Pt, thus maintaining the efficiency of the photodeposition process toward Pt single atoms.

To enhance our understanding of the experimental findings, we employ ab initio molecular dynamics (AIMD) simulations to investigate the dynamic behavior of Pt SAs under solvent conditions [45]. Radial distribution function (RDF, Figure 5f) analysis reveals a stepwise interaction process where HBr molecules approach the Pt site, ultimately forming Pt−HBr complexes. During these interactions, the Pt−HBr bond length progressively decreases, reflecting gradual bond strengthening. In addition, the Pt−Br_psk_ and Pt−I_psk_ bonds exhibit elongation compared to the initial Pt‐SA/MPBI configuration optimized under vacuum conditions. Specifically, the first coordination peak of the Pt−I_psk_ bond shifts from 2.54 to 2.78 Å, while the corresponding Pt−Br_psk_ bond peak shifts from 2.47 to 2.54 Å. These coordinated structural changes illustrate the solvent‐mediated modulation of Pt coordination environments, where HBr interaction not only stabilizes the Pt−HBr complex but also weakens the original Pt−halide coordination network. Such changes of both Pt−I_psk_ and Pt−Br_psk_ bonds suggest a displacement of Pt SAs, due to the dynamic atomic interactions occurring within the system. Pt SAs are not fixed in a rigid lattice structure, but are rather subject to positional fluctuations, leading to a weakening in the anchorage of Pt SAs upon HBr adsorption. Dynamic simulation calculations were initiated to examine the actual bond breaking of Pt−I_psk_ and Pt−Br_psk_ bonds but were prematurely halted due to the extensive computational time required. Nonetheless, a clear trend toward bond destabilization is evident in the dynamic solid‐liquid interface environment, consistent with the density functional theory (DFT) findings. The manipulation of single Pt atom dynamics is inspired by the adaptable characteristics of dynamic covalent bonds in polymer science, which permit reversible interactions and significant structural flexibility. Such adaptability enables these materials to modify their form or substance in response to varying environmental signals. Applying these principles to SACs leads to dynamic single‐atom self‐healing on halide perovskites, ensuring their structural restoration and consistent efficacy.

The practical implementation and extensibility of the adaptive concept rely on two essential and interconnected conditions: a dynamically restructuring photoactive host, and a single‐atom metal selected based on appropriate thermodynamic and bonding criteria. The substrate needs to be an ionic crystal capable of a dynamic dissolution‐precipitation equilibrium in a saturated precursor solution, which provides a labile interface for the reversible exchange of anchored species. The dynamic restructuring, inherent to halide perovskites under these conditions, is crucial for the continuous release and re‐incorporation of metal sites. In addition, the host material should exhibit strong intrinsic photoactivity to generate the photoelectrons required for driving metal ion reduction. For the single‐atom metal, a sufficiently positive reduction potential is necessary to permit efficient photoreduction by the host's conduction‐band electrons, enabling its initial deposition. Subsequently, the stabilization of the anchored metal atom critically depends on the chemical nature of the bond formed with the surface halides. A small electronegativity difference between the metal and the halide (e.g., Br or I) favors electron sharing and reduces ionic character. This is further reinforced by effective orbital interactions, where overlap between the metal d‐orbitals and halide p‐orbitals facilitates both *σ*‐donation and *π*‐backbonding. These combined effects enhance covalent bonding, which is essential for resisting dissociation in the acidic halide medium. Experimental investigations, including XPS analysis, support this rationale (Figure S26). On MPBI, metals like Au that meet the criteria demonstrate reversible deposition and dissolution, a behavior not observed for metals such as Fe under the same photodeposition conditions. Furthermore, we show that the core design principle can be successfully applied to other perovskite compositions, such as lead‐free MA_3_Bi_2_(Br_x_I_1−x_)_9_.

## 3 Conclusions

In summary, this work presents a photoresponsive platform that integrates SACs with dynamic halide perovskite supports, turning the traditional drawback of perovskite ion mobility into a functional mechanism for light‐regulated self‐repair and reversible atomic restructuring. We establish adaptive metal–support interactions enabled by the soft ionic lattice of perovskites, utilizing electronic and atomic structure synergy through bandgap funneling engineering in SACs. In contrast to conventional dynamic SACs that require energy‐intensive redispersion from clusters, our approach employs a continuous “bottom‐up” mechanism for atomic deposition under intermittent illumination, effectively preventing SA aggregation and ensuring long‐term stability. Integrated charge‐carrier dynamics and theoretical analyses show that the unique coordination structure and electronic characteristics of Pt‐SA/MPBIs enhance the photogenerated electron transfer from MPBI to Pt SAs, while reducing the Gibbs free energy and accelerating the kinetics of hydrogen halide splitting for hydrogen generation. The study advances atomic‐scale understanding of light‐controlled bond dynamics and demonstrates ionic lattices as versatile platforms for functional materials design.

## Conflicts of Interest

The authors declare no conflicts of interest.

## Supporting information




**Supporting File**: adma72725‐sup‐0001‐SuppMat.pdf.

## Data Availability

The data that support the findings of this study are available from the corresponding author upon reasonable request.
