# Supplementary material for: Photoresponsive Adaptive Reconfiguration of Single‐Atom Interface With Intermittent Light and Soft Ionic Lattices
Source: Adv Mater. 2026 Mar 9;38(19):e18557. doi: 10.1002/adma.202518557 (PMC13040514; doi:10.1002/adma.202518557)
Supplement: Supplementary file 1 — Supporting File: adma72725‐sup‐0001‐SuppMat.pdf. [file ADMA-38-e18557-s001.pdf]

Photoresponsive Adaptive Reconfiguration of Single-Atom Interface with Intermittent Light and Soft Ionic Lattices

Li Yu,[a] Jui-Cheng Kao,[b] Yuefeng Zhang,[c] Chun Hong Mak,[a] Yu-Chieh Lo,[b] Chun-Wei Pao,[d] Jyh-Pin Chou,[e] Zhenbin Wang,[ac] Ting-Shan Chan,[f] Hao Ming Chen,*[fg] and Hsien-Yi Hsu*[achi]

[*] [a] Dr. L. Yu, Dr. C. H. Mak, Prof. Z. Wang, Prof. H.-Y. Hsu
School of Energy and Environment, City University of Hong Kong, Kowloon Tong, Hong Kong, China
E-mail: sam.hyhsu@cityu.edu.hk
[b] Dr. J.-C. Kao, Prof. Y.-C. Lo
Department of Materials Science and Engineering, National Yang Ming Chiao Tung University, Hsinchu 30010, Taiwan
[c] Dr. Y. Zhang, Prof. Z. Wang, Prof. H.-Y. Hsu
Department of Materials Science and Engineering, City University of Hong Kong, Kowloon Tong, Hong Kong, China
[d] Prof. C.-W. Pao
Research Center for Applied Sciences, Academia Sinica, Taipei 11529, Taiwan
[e] Prof. J.-P. Chou
Graduate School of Advanced Technology, National Taiwan University, Taipei 106319, Taiwan
[f] Prof. T.-S. Chan, Prof. H. M. Chen
National Synchrotron Radiation Research Center, Hsinchu 30076, Taiwan
[g] Prof. H. M. Chen
Department of Chemistry & Center for Emerging Materials and Advanced Devices, National Taiwan University, Taipei 10617, Taiwan
E-mail: haomingchen@ntu.edu.tw
[h] Prof. H.-Y. Hsu
Center for Functional Photonics (CFP), City University of Hong Kong, Kowloon Tong, Hong Kong, China
[i] Prof. H.-Y. Hsu
Shenzhen Research Institute of City University of Hong Kong, Shenzhen 518057, China

Supplementary Methods

Preparation of $MAPbX_3$, $MAPb(Br_xI_{1-x})_3$, and their saturated solutions. $MAPbX_3$ was synthesized through its saturated HX acid solution, where X denotes a halogen such as bromine (Br) or iodine (I). A stoichiometric mixture of methylammonium halide (MAX) and lead halide (PbX_2) was dissolved in a 1:1 molar ratio into an HX acid solution under vigorous stirring. The solution consisted of either 57 wt% hydroiodic acid (HI) or 48 wt% hydrobromic acid (HBr) in water, supplemented with 20% (by volume) of a 50 wt% aqueous hypophosphorous acid (H_3PO_2) solution. Hypophosphorous acid was used to selectively reduce the I_3^- ions generated during the following photocatalytic hydrogen evolution reaction (PHER). The mixture was heated to 95 °C to ensure complete dissolution and maintained at this temperature for one hour before cooling naturally to room temperature. The resulting $MAPbX_3$ precipitate was then separated from the saturated solution.

To prepare $MAPb(Br_xI_{1-x})_3$, an excess of $MAPbBr_3$ powder was introduced into a mixed solution of HBr, H_3PO_2, and HI in a 7:2:1 volume ratio. Ultrasonic stirring was applied to ensure saturation of the mixture. The mixture underwent a light-assisted halogen ion exchange reaction by irradiation with visible light from a 300 W xenon lamp equipped with a 420 nm cutoff filter for 6 hours, providing an intensity of 80 mW cm^{-2} on the sample surface. The process was conducted at a constant 25 °C using a circulating water system. Subsequently, $MAPb(Br_xI_{1-x})_3$ precipitate was harvested and dried, and its saturated solution was collected.

Synthesis of $Pt/MAPb(Br_xI_{1-x})_3$ and $Pt/MAPbX_3$. $Pt/MAPb(Br_xI_{1-x})_3$ was produced via a light-assisted platinum loading technique. A homogeneous mixture was obtained by stirring 200 mg of $MAPb(Br_xI_{1-x})_3$ with an appropriate amount of $H_2PtCl_6{\cdot}6H_2O$ in a pre-prepared perovskite-saturated solution. The suspension underwent alternating cycles of vacuum exposure to visible light (6 hours) followed by darkness (3 hours), with other conditions similar to those used for the halide exchange reaction. The catalyst was collected either immediately after light exposure or after the dark field incubation. The resultant $Pt/MAPb(Br_xI_{1-x})_3$-saturated solution was employed for subsequent hydrogen evolution measurements. $Pt/MAPbX_3$ was synthesized using an analogous method, substituting $MAPbX_3$ and its saturated solution for $MAPb(Br_xI_{1-x})_3$ and its corresponding saturated solution.

Structure characterizations. X-ray diffraction (XRD) with a PANalytical X'Pert3 powder system and Cu Kα radiation was used for crystal structure analysis. Transmission electron microscopy (TEM) and high-resolution high-angle annular dark field scanning transmission electron microscopy (HAADF-STEM) images were captured on a double aberration-corrected JEOL JEM-ARM300F microscope. To mitigate electron beam-induced degradation, alignments and focusing were conducted on regions separate from those being imaged. For chemical analysis, X-ray photoelectron spectroscopy (XPS) data was collected with a Thermo Scientific K-Alpha$^+$ spectrometer, utilizing monochromatized Al Kα radiation and an Ar^+ cluster ion gun. The sputtering process used an Ar^+ ion beam at 3.0 keV energy, and the calibration of elemental peak positions was based on the C 1*s* reference at 284.8 eV. Surface chemical characterization was conducted using a time-of-flight secondary ion mass spectrometer (ToF-SIMS V, IONTOF GmbH) equipped with a pulsed Bi_3^+ primary ion source. Measurements were performed under high vacuum with charge neutralization via a low-energy electron flood gun. Mass spectra were acquired across a 200 × 200 μm^2 analysis area in high mass resolution mode to ensure precise identification of molecular fragments and elemental species. Quantitative determination of platinum content was performed using inductively coupled plasma optical emission spectrometry (ICP-OES, PerkinElmer Optima 8300). Prior to analysis, the samples underwent heat-assisted digestion in a mixed-acid system to ensure complete dissolution. Solid-state optical properties were investigated through diffuse reflectance spectroscopy (DRS) using a PerkinElmer LAMBDA 1050+ spectrophotometer equipped with an integrating sphere. Spectral data were acquired across the UV-visible to near-infrared range with a resolution of 1 nm, using $BaSO_4$ powder as a 100% reflectance reference. UV-visible absorption measurements in solution phase were conducted using quartz cuvettes. Fourier transform infrared spectroscopy (FTIR) was conducted on a Shimadzu IRAffinity-1 spectrometer. Steady-state photoluminescence (PL) spectra were obtained using a Shimadzu RF-5301PC fluorescence spectrometer. The PL decay dynamics were investigated through time-resolved photoluminescence (TRPL) spectroscopy using a PicoQuant FluoTime 200 spectrometer, with the data acquisition managed by a time-correlated single-photon counting (TCSPC) system. The laser used for tests delivered pulses under 200 ps with an energy density of approximately 30 nJ cm^{-2}. To analyze the decay profiles, a biexponential

decay function was applied to deconvolute the sample's intrinsic response function.

Time-resolved pump-probe measurements were performed using a femtosecond transient absorption spectrometer (HELIOS, Ultrafast Systems). The experimental setup employed a femtosecond laser source operating at a central wavelength of 800 nm, with a repetition rate of 1 kHz and pulse duration of approximately 100 fs. The primary laser beam was split into two paths: The first path served as the pump beam, which was directed through an optical parametric amplifier (OPA, TOPAS-Prime, Light Conversion) to generate tunable excitation wavelengths spanning 260–2000 nm. The second path was focused onto a sapphire crystal to produce a broadband probe beam covering the spectral range of 400–800 nm. Within the experimental configuration for sample excitation, the probe beam was aligned perpendicular to the sample surface, while the pump beam was incident at an angle of approximately 45°, enabling resonant excitation conditions for transient absorption characterization. Temporal synchronization between the two beams was achieved using a motorized mechanical delay stage to precisely control their arrival times at the sample. Spatial overlap was ensured by focusing both beams to spot diameters of ~100 μm (pump) and ~50 μm (probe) at the sample plane.

X-ray absorption spectroscopy. X-ray absorption fine structure (XAFS) measurements were conducted at the Pt L_3-edge in fluorescence mode under standard conditions at the BL17C beamline of the Taiwan Light Source (TLS) and the BL14W1 beamline of the Shanghai Synchrotron Radiation Facility (SSRF). Each scan was calibrated for energy using a Pt foil (11.564 keV), which helped to rectify any discrepancies in incident energy. The XAFS spectra were processed in the Athena software including energy calibration, averaging, and background subtraction. The normalization of the XAFS spectra was carried out using a consistent approach that involved a baseline adjustment through linear regression within the pre-edge region, complemented by a quadratic background correction in the post-edge phase. The $\chi(k)$ data was transformed into corresponding R-space spectra through Fourier analysis, with a Hanning window function used for modulation.

The differentiation of backscattering elements from Pt in the extended XAFS (EXAFS) spectra was achieved through wavelet transform (WT) analysis. The qualitative assessment of the shell atoms' backscattering effects utilized the Morlet WT within the Igor Pro software, applying parameters of $\kappa = 6$, $\sigma = 1$ over a radial distance range ($R + \Delta R$) from 1 to 4 Å. The Morlet wavelet was chosen for its fast oscillatory part localized in a Gaussian envelope, which closely resembles the real and imaginary components of an EXAFS spectrum. A continuous wavelet transform was executed on 128 data points, followed by the generation of a 2D contour plot representing the projection of a 3D graph for visual interpretation.

The Artemis software was utilized for the final processing of EXAFS spectra data. Scattering paths were theoretically generated using FEFF6, and multiple path combinations were evaluated during the fitting process to select the most suitable ones. The amplitude reduction factor (S_0^2) was determined by fitting a Pt foil with a pre-established coordination number, which then served as a reference for the fitting of subsequent samples. The number of independent data points (N_{ind}) was calculated using the formula $2\Delta k\Delta R/\pi$, where Δk and ΔR represent the fitted ranges in k- and R-space, respectively. Both the magnitude and real part of the Fourier-transformed EXAFS data, ranging from 1.5 to 3.5 Å, were subjected to fitting procedures. A Hanning window with a dk value of 1 was utilized during this process. Optimization was carried out across k-weights of 1, 2, and 3 to derive quantitative structural parameters associated with the central atoms. These parameters included scattering amplitudes, interatomic distances, phase shifts, and Debye-Waller factors. The quality of the curve fitting was quantified using an R factor, defined as $\Sigma(\chi_{data} - \chi_{fit})^2/\Sigma(\chi_{data})^2$, which was consistently maintained below 0.02 to ensure accuracy.

Electrochemical and photoelectrochemical characterizations. The electrochemical and photoelectrochemical behavior was documented using a CHI760E electrochemical workstation. This setup involved a standard three-electrode system comprising a working electrode, an Ag/Ag^+ reference electrode (silver wire in 0.01 M $AgNO_3$ in acetonitrile), and a platinum foil counter electrode in dichloromethane containing 0.1 M tetrabutylammonium hexafluorophosphate ($TBAPF_6$). Calibration of the reference electrode was performed using a ferrocene internal standard. The working electrode was prepared by dispersing the catalyst powder in dichloromethane to create a uniform ink, which was then pipetted onto a polished clean glassy carbon electrode (GCE) and allowed to dry. The photoelectrochemical experiments were conducted using the working electrode with the catalyst-coated pre-cleaned FTO glass and a 300 W xenon lamp

equipped with a 420 nm cut-off filter as the visible light source. Electrochemical impedance spectroscopy (EIS) was measured at the open circuit potential, with a 10-mV amplitude over a frequency range of 100 kHz to 0.1 Hz.

Photocatalytic measurements. The assessment of photocatalytic hydrogen evolution activity was conducted within an irradiation chamber connected to a hermetically sealed gas circulation system. A 300 W xenon lamp with a 420 nm cut-off filter provided visible light illumination of an intensity of 80 mW cm^{-2} on the sample surface, as measured by a hand-held optical meter. In a typical experiment, 200 mg of the catalyst was dispersed in the corresponding perovskite saturated HX solution under constant stirring. The evolved hydrogen was quantified using a Shimadzu GC-2010 Plus gas chromatograph, using argon as the carrier gas. The photocatalyst's stability was evaluated through a series of cyclic photocatalytic tests, with each cycle lasting 6 h of visible light illumination followed by 3 h of dark incubation and interspersed with re-evacuation of the system. The visible-light-driven hydrogen evolution performance of the synthesized photocatalyst is further benchmarked against Pt-modified perovskite composites reported over different periods,[S1-13] as illustrated in Figure 4e.

The solar HI splitting efficiency is quantified as the fraction of solar energy harnessed to cleave the chemical bonds of HI. This efficiency metric is derived from the established redox potentials for iodide oxidation and proton reduction. The standard redox potentials for H^+/H_2 and I^-/I_3^- are 0 and 0.53 V (versus SHE). Based on the Nernst equation and the corresponding ion concentrations in the mixed HBr/HI/H_3PO_2 acid solution (8.8 M of H^+, 0.76 M of I^-, and 5.4×10^{-5} M of I_3^-), the redox potentials for H^+/H_2 and I^-/I_3^- in the current photocatalytic system are determined to be 0.056 and 0.414 V versus SHE, respectively. The potential (E) for HI splitting herein is calculated as 0.358 V. The solar HI splitting efficiency (η) is therefore estimated from the following equation:

$$\eta = [\text{Evolved } H_2 \text{ (mol)} \times 6.02 \times 10^{23} \times 2 \times 0.358 \times 1.6 \times 10^{-19}] / [P_{solar} \text{ (W)} \times \text{time (s)}]$$

The apparent quantum yield (AQY) for H_2 production was measured under monochromatic light produced through bandpass filters with different central wavelengths. AQY value is estimated from the following equation:

AQY = 2 × the number of produced hydrogen molecules / the number of incident photons

Computational methods. Structural optimization and ab initio molecular dynamics (AIMD) simulations were conducted via density functional theory as implemented in the Vienna ab initio Simulation Package (VASP). The Perdew–Burke–Ernzerhof functional, within the generalized gradient approximation, was employed to characterize electronic exchange and correlation effects.[S14] Core and valence electron interactions were modeled using the projector augmented wave approach,[S15,S16] with a plane-wave basis energy cutoff set at 400 eV for structural relaxation.[S17,S18] Energy and force convergence thresholds were established at 0.01 eV $Å^{-1}$ and 10^{-5} eV, respectively. To address van der Waals forces in water molecules and between water and substrates, the PBE-D3 method was applied.[S19] A 2 × 4 × 1 Monkhorst-Pack grid facilitated Brillouin zone sampling. Gibbs free energy changes for elementary reactions were computed employing the computational hydrogen electrode (CHE) method, as introduced by Nørskov and colleagues,[S20] which considers the variations in adsorption energy (ΔE), zero-point energy (ΔZPE), and entropy (ΔS) at a standard temperature (T), following the equation: $\Delta G = \Delta E + \Delta \text{ZPE} - T\Delta S$. The preoptimized Pt-SA/MPBI structure was immersed in a simulation box containing explicit HBr aqueous solvents and subjected to AIMD simulations within a canonical (NVT) ensemble. The simulations, with a time step of 0.5 fs, employed the Nose-Hoover thermostat to sustain a system temperature of 298 K. Charge neutrality in the system was maintained by balancing net electronic charges with ionic charges in the implicit solvent model, with compensatory charges distributed according to the Poisson–Boltzmann equation, a feature integrated into VASP as VASPsol.[S21]

Supplementary Figures

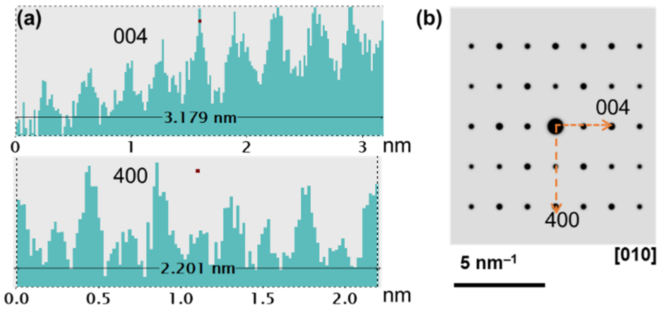

Figure S1. (a) Line intensity profiles of (004) and (400) interplanar spacings of MPBI as revealed in the AC-STEM image. (b) Simulated electron diffraction pattern of MPBI along the [010] zone axis.

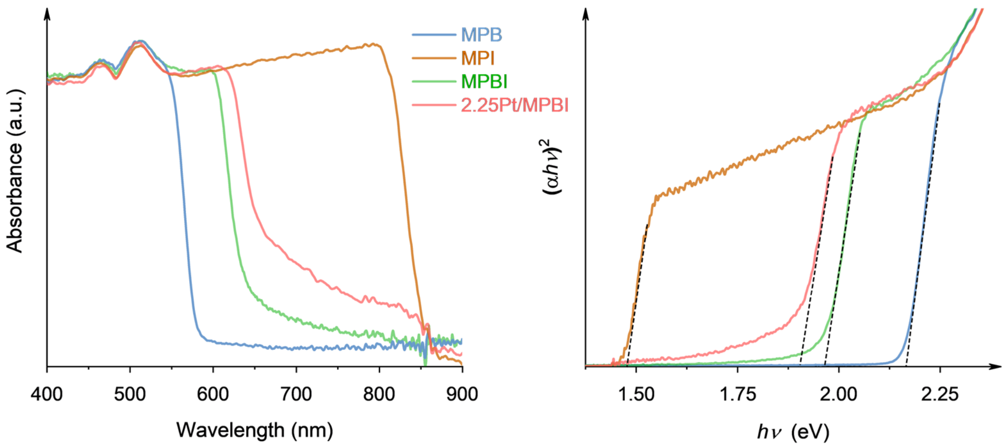

Figure S2. UV–vis diffuse reflectance spectra and direct-bandgap Tauc plots of the perovskites. The energy is determined from the wavelength of the absorbance spectrum.

The bandgap funnel effects and the introduction of Pt single atoms in MPBI induce electronic structure changes, closely related to the optical absorption properties. From the converted Tauc plots, the direct band gap of MPI, MPBI, and MPB are determined to be 1.48, 1.96, and 2.16 eV, respectively. The observation indicates a partial replacement of Br^- in MPB with I^- to form MPBI. Due to I^- introduction, the absorption tail of MPBI covers red-light and near-infrared regions. 2.25Pt/MPBI maintains the fundamental optical absorption profile of pristine MPBI, while exhibiting a modest red shift in the absorption edge and a reduced band gap of 1.90 eV. The primary perovskite structures are well preserved after the deposition of Pt atoms, with slight structural disturbance on the local electronic structure and intrinsic optical band gap.

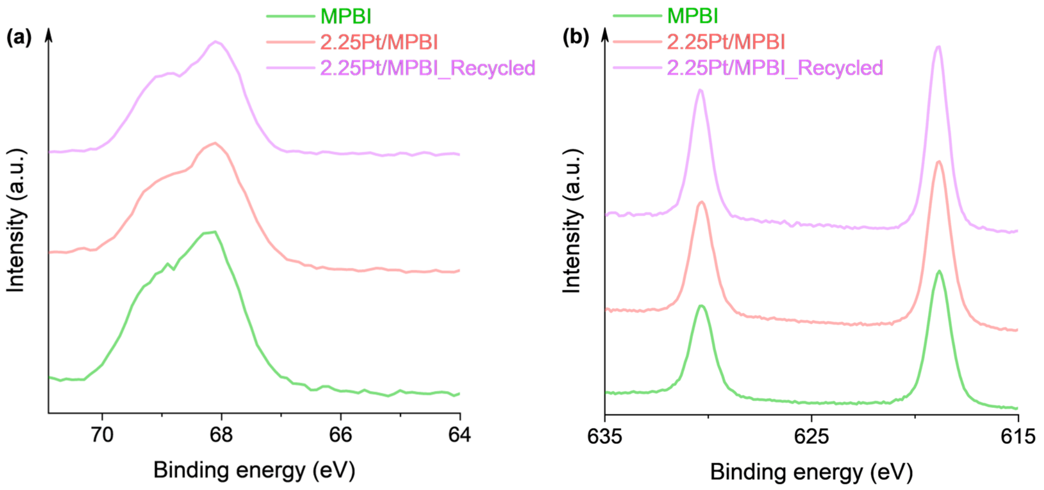

Figure S3. High-resolution XPS (a) Br 3d and (b) I 3d profiles. The surface Br/I ratio stabilizes following an initial adjustment period, showing that halide exchange attains kinetic balance. This stabilization preserves the necessary surface-to-bulk compositional gradient for maintaining the bandgap funneling effect.

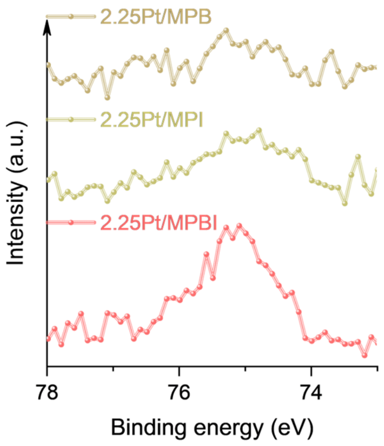

Figure S4. High-resolution XPS Pt $4f_{5/2}$ profiles.

An overlap in binding energy exists between the Pt $4f_{7/2}$ and Br $3d_{3/2}$ peaks. Given the substantially higher bromine content, the Br $3d_{3/2}$ signal dominates this spectral region, making direct analysis of the Pt $4f_{7/2}$ peak unreliable. However, the XPS spectrum clearly reveals the characteristic spin-orbit splitting of the Pt 4f region, exhibiting a well-defined doublet structure with the theoretical 4:3 intensity ratio between the Pt $4f_{7/2}$ and Pt $4f_{5/2}$ peaks. Furthermore, the binding energy separation between these Pt peaks remains invariant for each oxidation state. The consistent energy separation and peak area ratio demonstrate a direct correlation between these two Pt peaks. This correlation allows us to accurately determine the chemical state of Pt through analysis of the isolated Pt $4f_{5/2}$ peak, which remains free from spectral interference.

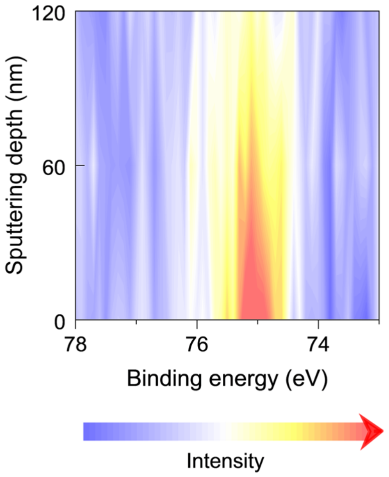

Figure S5. Depth profile analysis of background-corrected Pt $4f_{5/2}$ XPS spectra for 2.25Pt/MPBI, visualized through color-mapped intensity contours.

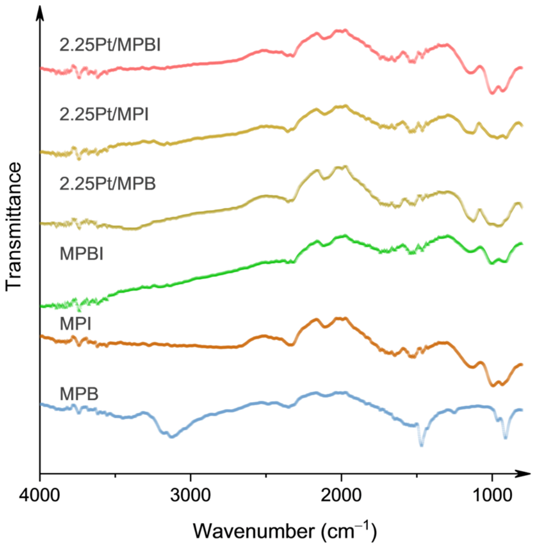

Figure S6. FTIR spectra.

Following the symmetry analysis of the space group, we can describe the internal vibrations of the MA molecule using the symmetry species A1, A2, and E, which correlate with 12 fundamental frequencies (*v*1−*v*12).[S22] These fundamental frequencies are assigned to the MA stretching, bending, rocking, torsional oscillation, and the C–N stretching modes. Specifically, the vibrations include two symmetric A1 modes (*v*1-stretching and *v*3-bending), two asymmetric double-degenerated E modes (*v*7-stretching and *v*9-bending) related to NH_3, and two symmetric A1 modes (*v*2-stretching and *v*4-bending) and two asymmetric double-degenerated E modes (*v*8-stretching and *v*10-bending) associated with CH_3. In addition, there is an A1 mode (*v*5, C–N stretching), one MA torsional oscillation A2 mode (*v*6), and two double-degenerated E modes (*v*11 for NH_3 and *v*12 for CH_3) that are associated with MA rocking modes.

The Fourier transform IR confirm the stable presence of $MAPbX_3$, as all samples record similar characteristic frequencies of CH/NH rocking (*v*12 at ~916 cm^{-1}, *v*11 + *v*12 at ~2120 cm^{-1}), C–N stretching mode (*v*5 at ~1000 cm^{-1}), and CH/NH bending modes (*v*3 at ~1460 cm^{-1}, and *v*9 at ~1545 cm^{-1}) of the MA molecule.

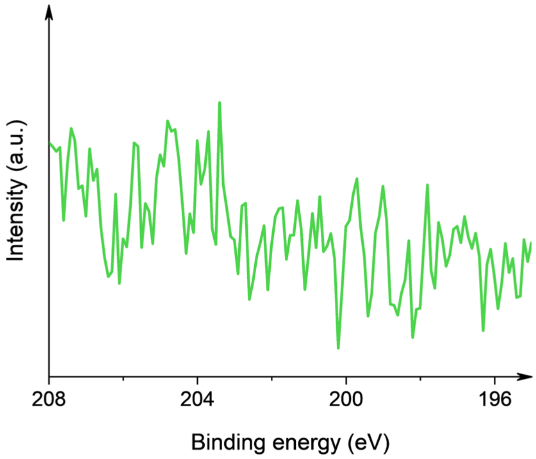

Figure S7. High-resolution XPS Cl 2p profiles of 2.25Pt/MPBI.

A key factor for suppressed Cl^- incorporation is the significant concentration difference in the synthesis medium. The Cl^- ions introduced by $H_2PtCl_6{\cdot}6H_2O$ are present at only about 4.6 mM, whereas the concentrations of Br^- and I^- ions are substantially higher—approximately 7.5 M and 0.76 M, respectively. The orders-of-magnitude excess of Br^-/I^- thermodynamically disfavors the exchange of these lattice halides with the trace amount of Cl^-. Kinetic factors related to exchange dynamics further contribute to the limited Cl^- incorporation into Br^-/I^--based perovskites. As indicated in prior studies,[S3] I^- in solution can rapidly exchange with Br^- at the $MAPbBr_3$ surface under light irradiation, a process likely facilitated by enhanced I^- ion migration within halide perovskites. In contrast, Cl^- has a lower halide exchange capability compared to I^-.[S23] XPS analysis further provides direct confirmation that Cl^- species are absent in the final composite.

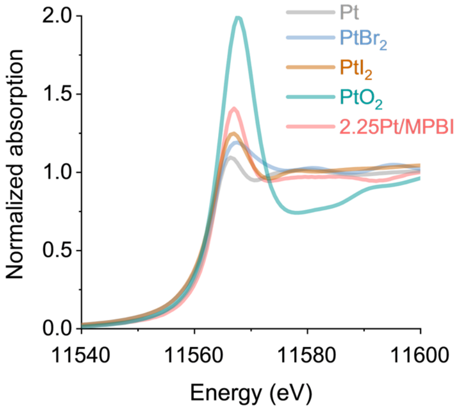

Figure S8. (a) Normalized XANES spectra for Pt L_3-edge.

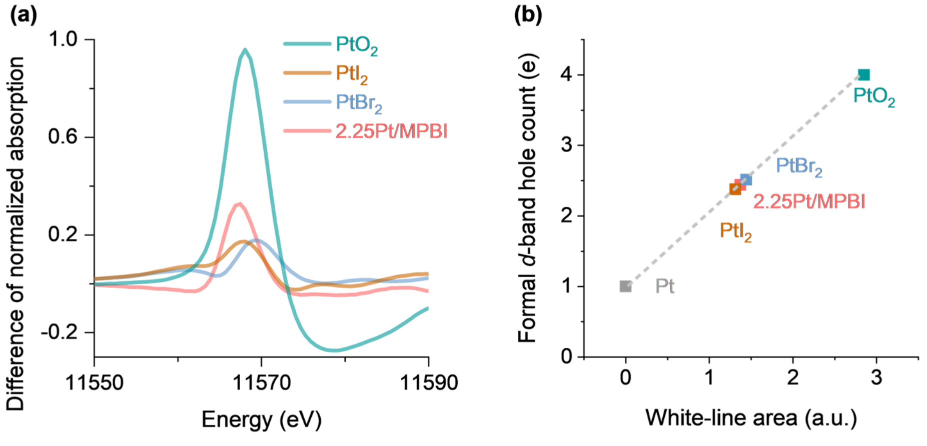

Figure S9. (a) Normalized ΔXANES spectra for Pt L_3-edge using Pt foil as the reference. (b) Fitted average formal *d*-band hole counts of Pt from ΔXANES spectra, using data from Pt^0 foil ($5d^96s^1$) and $Pt^{IV}O_2$ ($5d^66s^0$) as standards.

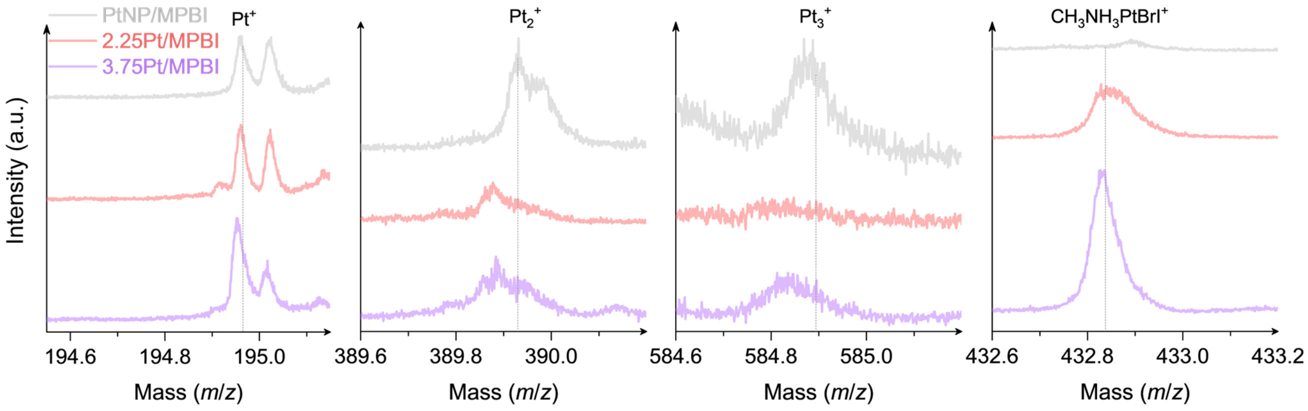

**Fragment ions**	**Pt^+**	**Pt_2^+**	**Pt_3^+**	**$CH_3NH_3PtBrI^+$**
m/z	194.965	389.930	584.894	432.838

Figure S10. Assignment of ToF-SIMS fragment ions: Distributions and theoretical *m/z*.

The peaks corresponding to Pt^+, Pt_2^+, Pt_3^+, and $CH_3NH_3PtBrI^+$ show slight mass shifts from their theoretical values. Such minor deviations are typical in ToF-SIMS analysis, especially for ions in higher mass ranges. They commonly result from several factors, such as non-linearities in the mass scale calibration at higher *m/z*, local variations in surface potential across the analysis area, and subtle differences in the initial kinetic energy distribution of larger, more complex molecular ions compared to atomic ions. The remaining unassigned peaks, particularly those near the Pt^+ signal, are mainly due to background species and common laboratory contaminants. These likely include widespread silicones, phthalate plasticizers, and residual species from substrates or solvents used in the sample preparation. Their presence is consistent with the extreme surface sensitivity of the ToF-SIMS technique.

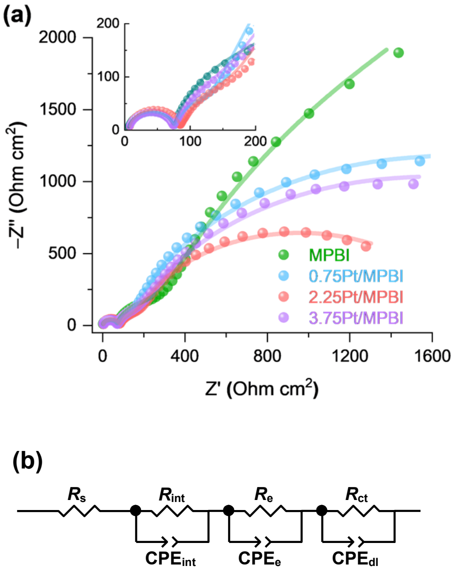

Figure S11. (a) Nyquist plots from electrochemical impedance spectroscopy (EIS), showing experimental data (solid spheres) and fitted spectra (solid lines). (b) Equivalent circuit model used for EIS data fitting.

The impedance spectral data are interpreted using an equivalent circuit model that reflects their observed characteristics. Semicircular patterns in Nyquist plots are typically modeled using a resistor paired with a capacitive component. Surface irregularities in electrodes often lead to deviations from ideal capacitive behavior at the solid-electrolyte interface. To account for this non-ideality, constant phase elements (CPEs) replace standard capacitors in the circuit.[S24] The impedance of a CPE is expressed mathematically as $Z_{CPE} = 1/(j\omega)^n Y_0$, where Y_0 (in Ω^{-1} cm^{-2} s^n) represents admittance of CPE and incorporates capacitance-related information, while n (ranging 0–1) serves as an ideality factor. Here, ω denotes angular frequency and j the imaginary unit. Depending on the n value, CPEs can simulate resistors, capacitors, inductors, or Warburg diffusion effects.

Experimental spectra closely match fitted curves, with fitting parameters detailed in Table S2. The high-frequency intercept on the real axis corresponds to resistor R_s (7–9 Ω cm^2), reflecting the system's stable internal resistance across all configurations. Three distinct semicircular regions emerge in the Nyquist plot: a high-frequency arc, a mid-to-high frequency arc, and a low-to-mid frequency arc. These correspond sequentially to (1) resistance and associated capacitance at the electrode/electrolyte interphase layers (R_{int}, CPE$_{int}$), (2) electronic conduction within the cathode (R_e, CPE$_e$), reflecting the electronic properties of the material, and (3) Faradaic charge transfer processes (R_{ct}, CPE$_{dl}$).[S25] R_{int} shows minimal variation across systems, indicating stable charge conduction between the electrolyte and electrode interfaces. In contrast, R_e and R_{ct} exhibit significant differences. The incorporation of Pt single atoms markedly lowers R_e, demonstrating the establishment of an efficient electron/ion-conductive network. The most pronounced change occurs in R_{ct}, where 2.25Pt/MPBI exhibits the lowest charge transfer resistance (~1609 Ω cm^2), highlighting its superior interfacial kinetics.

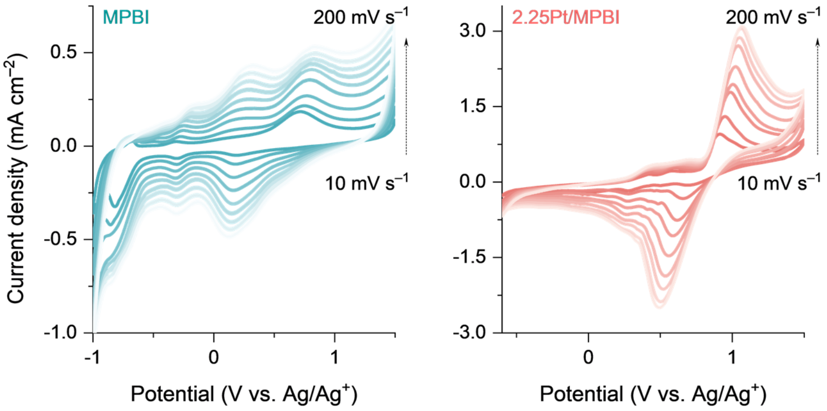

Figure S12. CV at different scan rates from 10 mV s^{-1} to 200 mV s^{-1}.

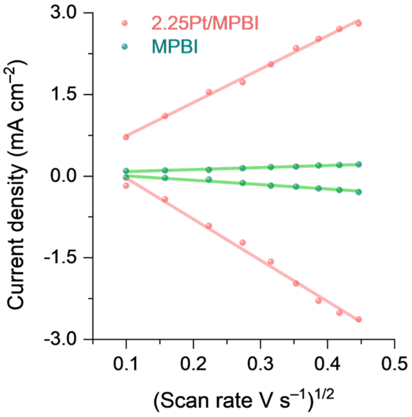

Figure S13. The linear relationship between i_p and the square root of scan rate.

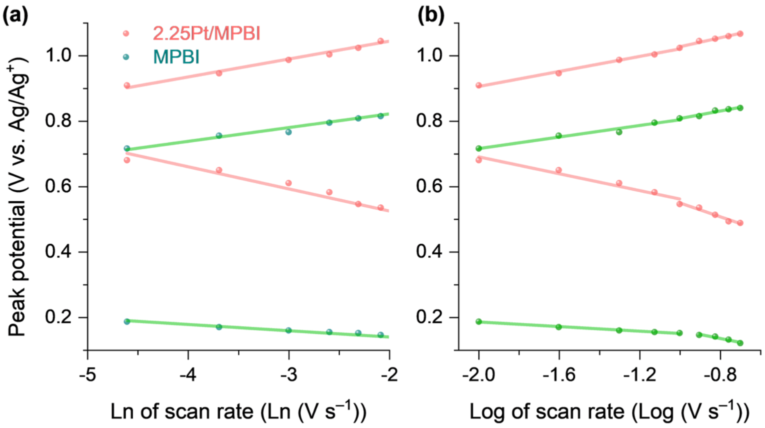

Figure S14. (a) The linear relationship between E_p and the natural logarithm of scan rate; (b) The plot of E_p against the log of scan rate at low and high scan rates for the anodic and cathodic branches.

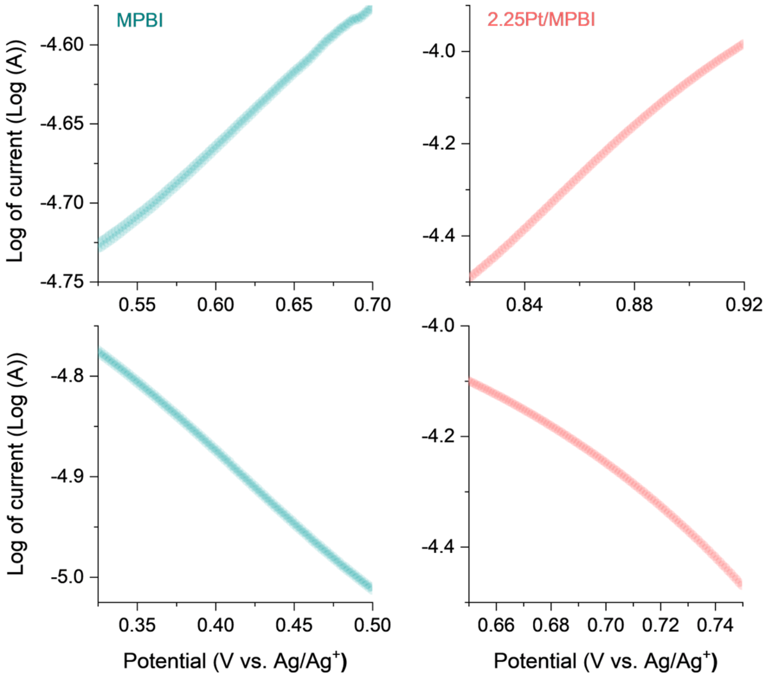

Figure S15. Tafel plots for the anodic and cathodic branches.

Cyclic voltammetry analysis provides comprehensive insights into the electrochemical reaction kinetics,[S26] as illustrated in Figure S12. The technique enables precise identification of oxidation and reduction zones through examination of potential variations induced by controlled system perturbations. At a scan rate of 10 mV s^{-1}, distinct anodic oxidation peaks emerge at 0.716 V and 0.909 V, with these characteristic potentials demonstrating a progressive positive shift as the scan rate increases. In addition, the cathodic reduction peak observed during the reverse scan exhibits a complementary negative potential shift, maintaining this trend up to the maximum tested scan rate of 200 mV s^{-1}. The non-unity reverse-to-forward peak current ratio (consistently differing from 1) provides compelling evidence for either chemically coupled reaction pathways or heterogeneous electron transfer processes operating with distinct kinetic rates within the system.

The fundamental relationship between electrochemical response and scan rate becomes evident through detailed analysis of peak current behavior. Both anodic and cathodic peak currents (I_p) exhibit a linear dependence on the square root of scan rate (Figure S13), a characteristic that confirms the diffusion-controlled nature of the electrode process. The observation shows agreement with the theoretical framework established by the Randles-Sevcik equation: $i_p = 2.69\times10^5 n^{3/2} C D^{1/2} v^{1/2}$, where the peak current density (i_p, A cm^{-2}) maintains a direct proportionality to the square root of scan rate (v, V s^{-1}), with the scaling factor encompassing three key physicochemical parameters: the number of electrons transferred in the electrochemical process (n), the bulk concentration of redox-active species (C, mol cm^{-3}), and their characteristic diffusion coefficient (D, cm^2 s^{-1}).

For quantitative determination of active substance concentration within the electrode, we performed additional cyclic voltammetry measurements at an ultra-low scan rate of 1 mV s^{-1}. This experimental approach allows accurate integration of EI/v across the entire potential window of interest, yielding the total faradaic charge associated with the electrode material's redox activity. The resulting charge quantification serves as the foundation for determining the quantity of electrochemically active species participating in the charge transfer processes.

Further kinetic analysis employs the Laviron equation to establish the relationship between peak potential (E_p) and the natural logarithm of scan rate (ln v), as presented in Figure S14a. The linear regression slope of this relationship, quantitatively expressed as $RT/(1-\alpha)nF$, contains critical information about the electron transfer coefficient (α) and the

number of electrons (n) exchanged during the electrochemical process. This relationship incorporates essential thermodynamic parameters including the universal gas constant (R), Faraday constant (F), and absolute temperature (T).

The electron transfer coefficient (α) receives additional verification through independent Tafel analysis based on the Butler-Volmer equation: $\log I_p = \log I_0 + (\alpha F/2.303RT)\eta$, where I_0 represents the exchange current and η denotes the overpotential. As predicted by this equation, the experimental data in Figure S15 demonstrate linearity between $\log I_p$ and applied potential, with the characteristic Tafel slope directly yielding the electron transfer coefficient through the relationship slope = $\alpha F/2.303RT$.

Complete kinetic characterization requires determination of the heterogeneous electron-transfer rate constant (k^0), achieved through application of the Gileadi method: $\log k^0 = -0.48\alpha + 0.52 + \log (nF\alpha V_c D/2.303RT)^{1/2}$. The critical scan rate parameter (V_c) in this equation emerges from analysis of the E_p vs. $\log v$ relationship across different scan rate regimes (Figure S14b), specifically identifying the intersection point that marks the transition between reversible and irreversible electrochemical behavior.

The kinetic parameter comparison presented in Table S3 reveals significant enhancements for the 2.25Pt/MPBI system relative to pristine MPBI, with all critical parameters (α, D, and k^0) showing notable improvement. These improvements demonstrate the crucial role of single-atom Pt incorporation in substantially enhancing both mass transport characteristics and heterogeneous electron transfer kinetics within the perovskite-based electrochemical system.

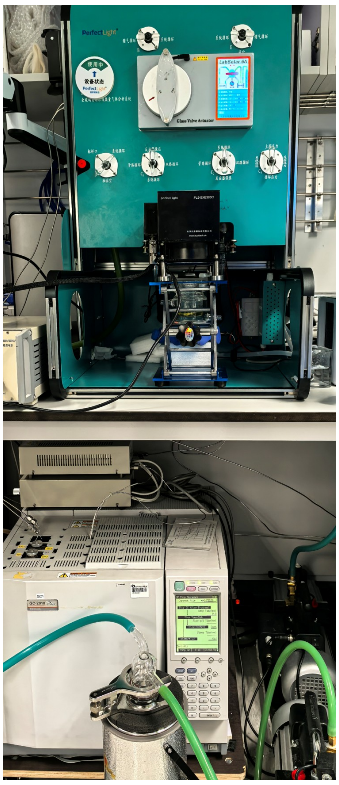

Figure S16. Integrated photocatalytic reaction system schematic with light source, reaction cell, gas flow path, and online gas analysis system.

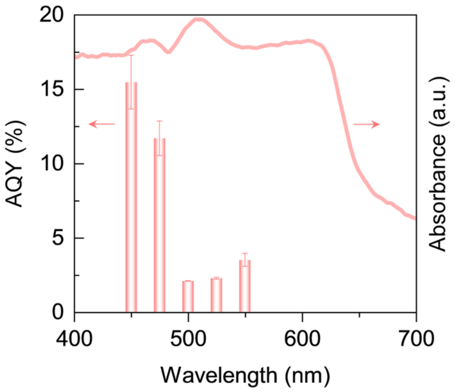

Figure S17. AQY calculation and UV–vis diffuse reflectance spectra for 2.25Pt/MPBI.

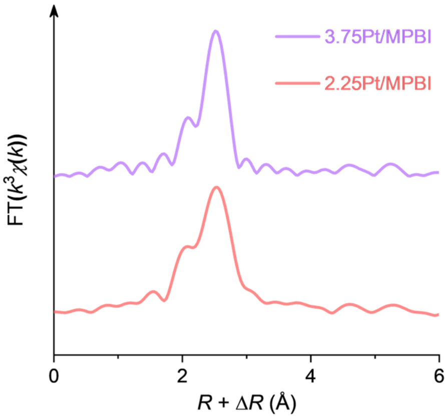

Figure S18. Fourier transform (FT) k^3-weighted $\chi(k)$-function of the Pt L_3-edge EXAFS spectra.

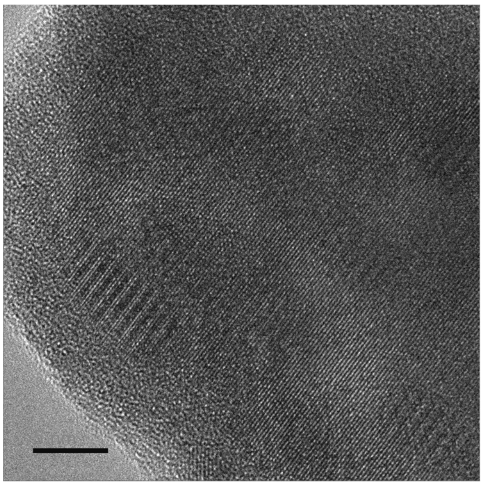

Figure S19. HRTEM image of 3.75Pt/MPBI (scale bar: 5 nm).

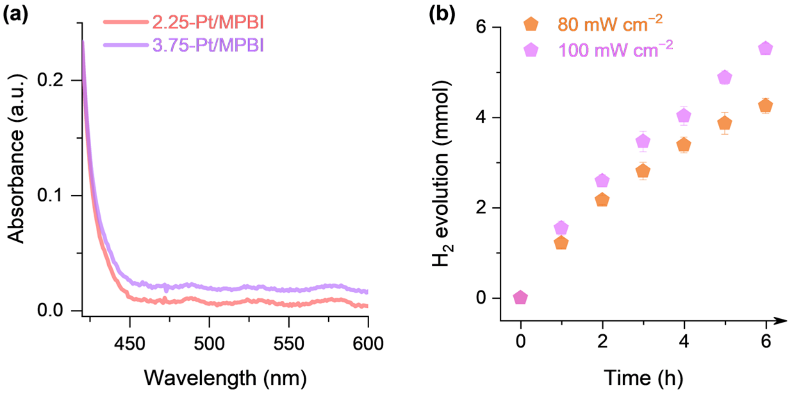

Figure S20. (a) Visible light absorption spectra of saturated solutions in different systems. (b) PHER with different illumination intensity for the 3.75Pt/MPBI system.

Our investigation into the Pt loading effect in Pt/MPBI catalysts reveals an intricate correlation between Pt content and PHER performance. The photocatalytic activity unexpectedly decreases when increasing the Pt loading from 2.25 wt% to 3.75 wt%, despite maintaining nearly identical Pt coordination environments (EXAFS analysis, Figure S18) and no visible nanoparticle formation (high-resolution TEM, Figure S19). ToF-SIMS detects predominant $CH_3NH_3PtBrI^+$ fragments in both samples, with higher intensity in 3.75Pt/MPBI (Figure S10). While trace amounts of Pt_2^+ and Pt_3^+ species show a slight increase in 3.75Pt/MPBI, indicating possible limited metallic aggregation, their low abundance relative to total Pt content suggests minimal impact on PHER activity. This observation confirms that Pt remains primarily stabilized through mixed halide coordination rather than extensive aggregation. The observed activity reduction in 3.75Pt/MPBI despite its predominant structural similarity to 2.25Pt/MPBI directs our attention to solution-phase photophysical effects. As Pt loading increases from 2.25 wt% to 3.75 wt%, the precursor solution exhibits progressively stronger visible light absorption (Figure S20a). The light absorption by dissolved Pt complexes in the solution attenuates photon flux reaching the catalyst surface, initiating a competing pathway that diminishes the effective light intensity available for photocatalytic reactions.

Using absorption at 450 nm as an example, the UV-Vis measurements in a 1 cm cuvette give absorbances of 0.0115 for the 2.25Pt/MPBI system and 0.0261 for the 3.75Pt/MPBI system. Applying the Beer–Lambert law to the actual ~3 cm optical path length in the reactor, the corresponding absorbance values are calculated as 0.0345 and 0.0783, respectively. These correspond to transmittances (T) of 92.4% and 83.5%. The light intensity reaching the photocatalyst surface differs by nearly 10% between the two systems. Although the raw absorbance values appear small, this difference represents a non-negligible reduction in photon flux under our standard illumination conditions. To directly test whether this attenuation causes performance decline, we conducted experiments with enhanced illumination intensity (Figure S20b). The compensatory effect of increased light intensity further indicates that differential light absorption is a key mechanistic factor behind the observed performance trend.

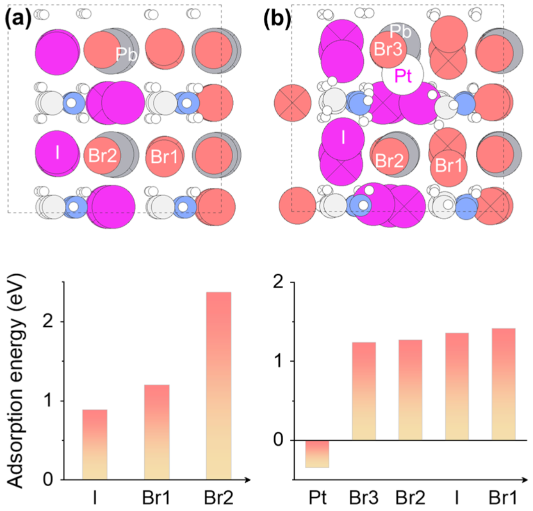

Figure S21. Adsorption sites and corresponding hydrogen adsorption energies for (a) MPBI and (b) Pt-SA/MPBI.

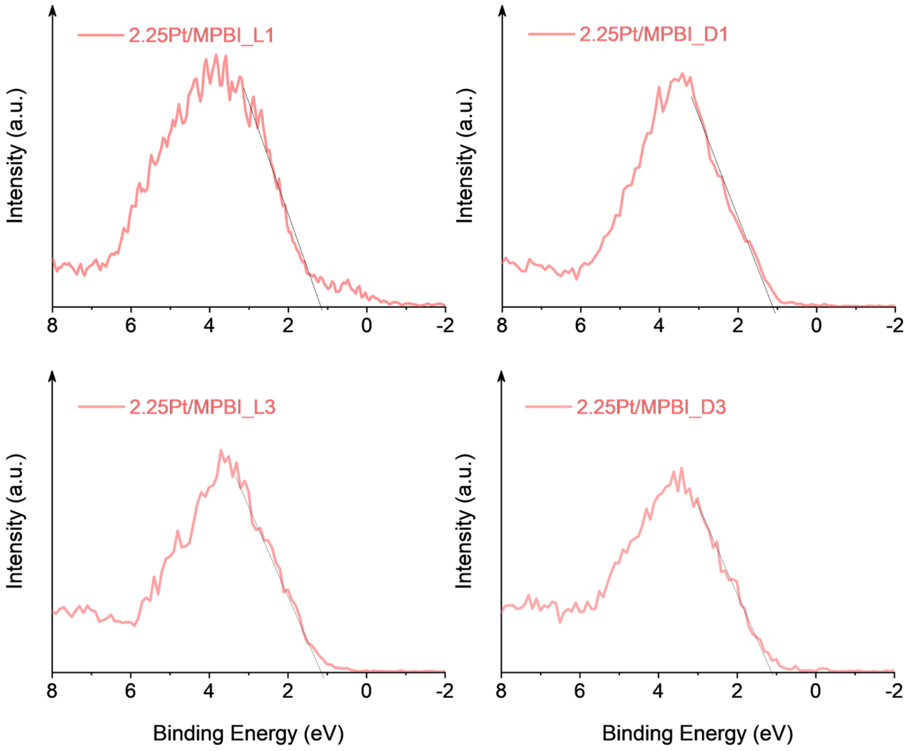

Figure S22. Comparison of the VBM energies of Pt/MPBIs.

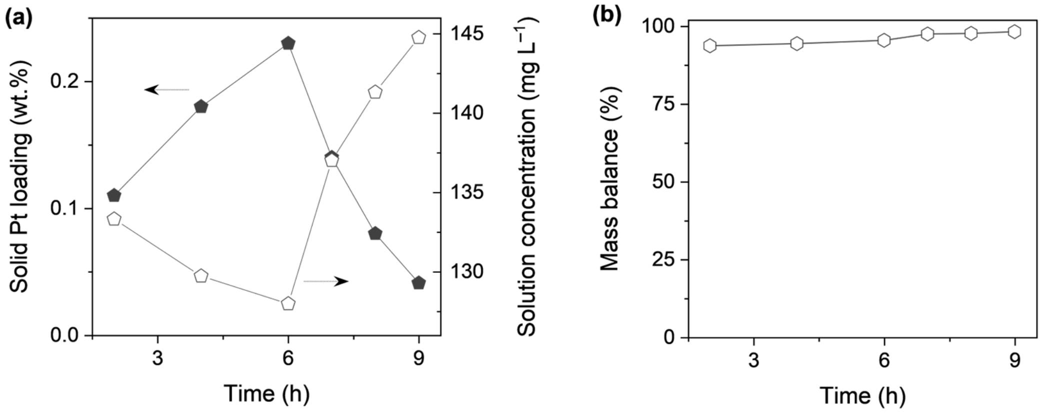

Figure S23. Pt mass balance in a programmed light–dark cycle based on ICP-OES measurements. (a) Evolution of Pt concentration in solution and surface content on perovskite. (b) Total Pt mass from both phases showing mass conservation throughout the cyclic experiment.

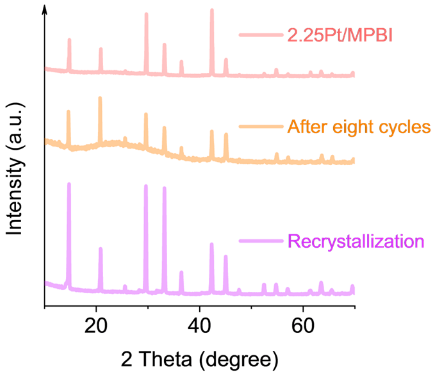

Figure S24. Structural stability evaluation via XRD analysis.

The perovskite phase itself remains intact, indicating no irreversible decomposition or phase segregation. The observed changes in diffraction intensity are associated with the dynamic and responsive character of the ionic perovskite lattice under reaction conditions. During extended cycling, continuous ion exchange and the local dissolution-reprecipitation processes inherent to the dynamic equilibrium can induce microstructural variations within the crystallites. These effects are known to influence relative peak intensities and may lead to slight peak broadening or shifts without changing the underlying crystal structure. These microstructural variations reflect the dynamic stability of the system rather than its degradation, and they do not compromise the catalytic hydrogen evolution performance, as supported by the consistent activity data. Moreover, a distinctive feature of our system is that such reversible microstructural changes can be reset. The perovskite-saturated solution environment enables a straightforward thermal dissolution and recrystallization step, which effectively restores the crystallinity. This demonstrates the robust and sustainable nature of the dynamic material platform.

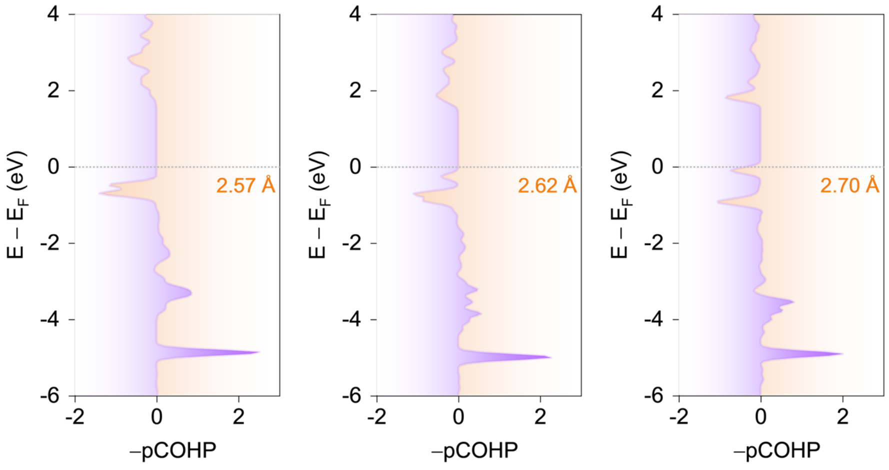

Figure S25. pCOHP analysis of the evolution of Pt−I$_{psk}$ bond strength with varying xHBr coverage (x = 0, 1, and 2, from left to right panels). The resultant bond lengths before and after adsorption are depicted in the accompanying figures.

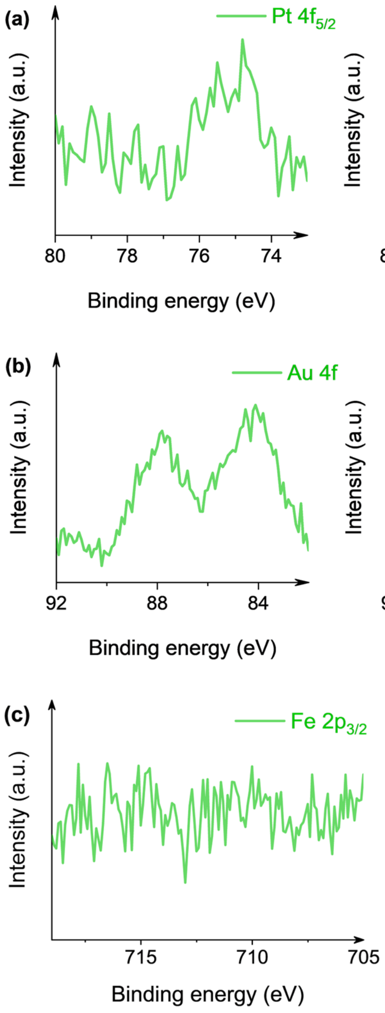

Figure S26. Probing the extensibility of the adaptive single-atom concept using XPS. (a) Pt $4f_{5/2}$ spectra for the lead-free $MA_3Bi_2(Br_xI_{1-x})_9$ system under light (left panel) and dark (right panel) conditions. (b) Au 4f spectra for the MPBI system under light (left panel) and dark (right panel) conditions. (c) Fe $2p_{3/2}$ spectra for the MPBI system under photocatalytic reaction conditions.

Supplementary Notes

Note S1. Stabilization of halide perovskites through dynamic dissolution-reprecipitation equilibrium

The system's stability is intrinsically linked to the dynamic dissolution-precipitation equilibrium established between the perovskite powder and its saturated precursor solution, a core concept elaborated in the foundational work by Park et al.[S1] As detailed in this study, the stability of the $MAPbX_3$ phase in an aqueous medium is not intrinsic but is critically dependent on the concentrations of key ions in solution. A key finding is that this phase remains stable only within a specific region of high ionic concentrations where the H^+ concentration meets or exceeds the halide ion concentration. In highly acidic media, the increased strength of hydrogen bonding between water molecules reduces their tendency to interact with and hydrate the perovskite crystal lattice. This effectively suppresses the formation of hydrated phases, thereby directing the ionic constituents toward the precipitation of the desired perovskite structure. The dynamic equilibrium itself is the primary mitigation strategy against the structural vulnerabilities. In this state, the perovskite surface is not static; it undergoes continuous, reversible exchange of ions (e.g., MA^+, PbI_3^-) with the saturated solution. This process provides a self-healing mechanism. Any localized dissolution or surface degradation is counterbalanced by the instantaneous reprecipitation of fresh perovskite material from the reservoir of ions in solution.

According to a similar principle, our system maintains the perovskite framework within its thermodynamically stable regime. This is achieved using concentrated hydrohalic acid (HBr/HI) solutions that are saturated with the corresponding perovskite components. Such a carefully controlled environment is essential to prevent the formation of competing hydrated perovskite phases or the irreversible decomposition into lead halide. The continuous regeneration inherent to this dynamic state preserves the structural integrity of the perovskite scaffold, as confirmed by XRD analysis (see Figure S24) and sustained photocatalytic hydrogen production over extended periods (e.g., 72 hours). The successful cyclic regeneration of Pt single atoms is consequently facilitated by, and dependent upon, this pre-established, stable perovskite-solution interface. Thus, stabilizing the perovskite framework and achieving dynamic metal anchoring are interrelated processes within a unified mechanism based on controlled dynamic equilibrium and ionic exchange.

Note S2. Dynamic stability of the halide gradient and bandgap funneling

Our analysis indicates that the Br/I gradient responsible for the bandgap-funnel structure undergoes dynamic exchange but remains functionally stable over extended cycling without homogenizing. The stability of this compositional gradient is governed by the significant concentration difference between halides in the system: the bromide concentration in solution is approximately an order of magnitude higher than that of iodide. As a result, the bulk of the perovskite host retains a Br-rich composition. Halide exchange is largely confined to the surface and near-surface regions, where ions are more labile. This process establishes a dynamic equilibrium rather than leading to a complete and irreversible homogenization. The continuous exchange is part of the material's inherent dynamic equilibrium with the saturated solution, a principle well established in prior work.[S1]

XPS analysis of the perovskite surface at different cycling stages directly supports the stabilized dynamic state. The data show that the surface Br/I ratio evolves initially but reaches a steady state after a period of reaction, indicating that surface halide exchange attains a kinetic balance (see Figure S3). This preserves a stable compositional gradient between the surface and the bulk, which in turn maintains the bandgap funneling effect. The persistence of this gradient is further supported by consistent photocatalytic performance. As shown in Figure 4d, the hydrogen evolution rate remains stable during long-term testing. Moreover, the efficiency of Pt photodeposition (detailed in Figure 5a) shows no significant decline over repeated cycles, and the VBM position is consistently maintained at ~1.1 eV below the Fermi level (Figure S22). The results collectively demonstrate that the dynamic reconfiguration of the halide gradient is self-limiting and converges to an equilibrium arrangement. This arrangement continuously sustains the bandgap funneling effect, which in turn facilitates the efficient and cyclic redeposition of Pt single atoms during the light-driven regenerative stages of our system.

Note S3. Dynamic deposition-dissolution equilibrium of Pt

In our experimental system, single Pt atoms are anchored to the perovskite surface through Pt–Br or Pt–I bonds, which are intrinsically labile in the perovskite-saturated HX solution (X = Br or I) we use. Under illumination, Pt deposition and its dissolution through bond cleavage co-occur, with the net deposition rate slightly exceeding that of dissolution. XPS and ICP analyses (Figures 5a and S23) confirm that even under light, a significant portion of the Pt precursor remains in the solution phase. It is important to clarify that the system is not designed to force the majority of Pt to deposit permanently onto the solid.

A key finding is that only a trace amount of deposited Pt is sufficient to significantly enhance the hydrogen evolution performance while preserving the structural integrity of the perovskite host. In this closed system, Pt is not consumed; it is continuously redistributed between the perovskite surface and the solution. The dissolution of Pt does not imply catalyst deactivation. Instead, it is an intentional feature of our design that makes possible the catalyst regeneration via the subsequent light-induced reduction and redeposition of Pt from the solution. The concept originated from our experimental observation that a prolonged dark rest period leads to improved photocatalytic performance in the following light cycle. This suggests that a dynamic dissolution-redeposition cycle refreshes the active sites. In an ideal scenario, the designed reversible dynamic process could theoretically be maintained, enabling continuous system regeneration across periodic light-dark cycles. The ultimate lifetime of the system is therefore not determined by irreversible Pt loss but by maintaining this dynamic balance.

Note S4. Photoresponsive adaptive single-atom interfaces: Conceptual design principles and extensibility

The practical application of this self-adaptive single-atom interface concept relies on two key conditions: a dynamically restructuring photoactive host, and the selection of metals based on appropriate thermodynamic and bonding criteria.

Dual functional requirements of the substrate material

A host material that supports a dynamic, light-regulated single-atom interface must simultaneously satisfy two key criteria. First, it must exhibit a reversible precipitation-dissolution equilibrium in a saturated precursor solution. This behavior relies on a crystalline framework held together by relatively labile ionic bonds, enabling continuous exchange of constituent ions between the solid surface and the solution. Halide perovskites in their saturated solutions naturally show such dynamic surface restructuring, which facilitates the release of anchored metal atoms back into the medium, forming a dynamic reservoir. Second, the host itself must possess intrinsic and efficient photoactivity. This is a fundamental requirement for it to be capable of generating photoexcited electrons with sufficient reducing power to drive the stepwise reduction of metal complexes from the solution onto its surface. Halide perovskites meet this requirement well due to their excellent light-harvesting and charge-separation properties.

Thermodynamic and bonding criteria for metal selection

Extending the concept to other metals involves additional constraints that are largely governed by thermodynamics and bond stability. Two combined criteria determine whether a given metal can form a stable, dynamically anchored single-atom site under our experimental conditions.

Thermodynamic feasibility of photoreductive deposition. The effective photoreduction and deposition of single atoms (SAs) require that the photogenerated electrons from the perovskite possess adequate reducing potential. Upon partial reduction, the electron cloud at the SA center becomes more diffuse and polarizable. The resulting excess electrons readily delocalize into the SA–X bonding region and populate the antibonding orbitals of the ligands. The significantly enhanced overlap and sharing of electron density between SA and X increases both the stability and covalent character of the SA–X bond, mitigating SA dissociation in solution. In the present composite, the photoactive surface is

predominantly $MAPbI_3$, with a conduction-band minimum around –0.5 V vs. SHE.[S27,S28] Standard reduction potentials of relevant metal-ion/metal couples are critical in this regard. The potentials for several first-row transition metals are: Ni^{2+}/Ni (–0.257 V), Co^{2+}/Co (–0.28 V), Fe^{2+}/Fe (–0.447 V), and Mn^{2+}/Mn (–1.185 V). These values are close to or more negative than the reducing power available from the perovskite's conduction band, making efficient photoreduction and deposition unlikely. Moreover, in the acidic halide solution used, these metals also compete unfavorably with the high concentration of H^+ ions, both thermodynamically and in terms of concentration. In contrast, reduction potentials for noble metal complexes like $[PtCl_4]^{2-}$/Pt (0.755 V) and $AuCl_4^-$/Au (1.002 V) are substantially more positive, which allows them to be readily reduced by the photoexcited electrons.

Stability of the metal-halide bond in a concentrated acidic halide medium. In the concentrated HBr/HI medium, the bond anchoring the single metal atom to the perovskite surface must resist rapid ionic dissolution. The electronegativity difference ($\Delta\chi$) between the metal and the halide (Br or I) serves as a useful indicator. Pauling electronegativities are: Br (2.96), I (2.66), Pt (2.28), Au (2.54), Ni (1.91), Co (1.88), Fe (1.83), Mn (1.55). The small $\Delta\chi$ between Pt and I (~0.38) or Br (~0.68) favors covalent bonding, which contributes to stability. Moreover, the close energy match and compatible symmetry between the Pt 5d orbitals and the halogen 4p (Br) or 5p (I) orbitals promote efficient orbital overlap. This interaction facilitates strong σ-coordination, where lone-pair electrons from the halogen donate into vacant hybrid orbitals of Pt, as well as significant π-backbonding from filled Pt d-orbitals into empty π-symmetric acceptor orbitals on the halogen (such as vacant d-orbitals or σ^* antibonding orbitals). The synergistic σ-donation and π-backbonding significantly enhance the covalent character and strength of the bond. In contrast, the $\Delta\chi$ value between halogens (Br/I) and first-row transition metals is considerably larger, for instance, ~1.13 for Fe–Br. This indicates a more ionic character, which in an acidic halide solution would promote dissociation and thus prevent stable anchoring of the metal.

Following these principles, metals such as Au, which shares similar reduction potential and bonding characteristics with Pt, are also suitable for constructing a dynamically anchored single-atom system. As shown by the XPS data in Figure S26, Au atoms exhibit reversible changes on the MPBI surface under alternating light and dark conditions. In contrast, Fe does not undergo efficient photodeposition under the same conditions. Moreover, the same design principle for a dynamically anchored Pt single-atom catalyst can be extended to other halide perovskite hosts, such as the lead-free $MA_3Bi_2(Br_xI_{1-x})_9$ perovskite.

In summary, the present system relies on a combination of a sufficiently positive reduction potential and covalent metal-halide bonding-criteria that noble metals like Pt and Au satisfy but most earth-abundant first-row transition metals do not, at least within the current host. Adapting the concept to other metals would likely require substantial modifications, such as using a different perovskite composition to adjust the band structure (for better reducing power) or the surface halide identity (to modulate bond ionicity).

Supplementary Tables

Table S1. Fitting parameters of biexponential decay function for TRPL kinetics.*

**Photocatalysts**	τ_1 **(ns)**	A_1	τ_2 **(ns)**	A_2	τ_{avg} **(ns)**
MPBI	3.366	0.807	14.04	0.193	5.426
0.75Pt/MPBI	2.293	0.836	5.879	0.164	2.881
2.25Pt/MPBI	2.057	0.912	6.616	0.088	2.458
3.75Pt/MPBI	1.970	0.871	6.786	0.129	2.591

*In biexponential TRPL analysis, τ_1 and τ_2 denote the characteristic times for distinct recombination pathways. The relative contributions of these pathways are quantified through their amplitude coefficients (A_1 and A_2), which represent the proportion of photogenerated carriers participating in each recombination channel.

Table S2. Fitted parameters of the equivalent circuit model for EIS spectra (values in parentheses indicate fitting errors).

**Parameters**		**MPBI**	**0.75Pt/MPBI**	**2.25Pt/MPBI**	**3.75Pt/MPBI**
R_s/Ω cm^2		7.29 (0.0985)	8.69 (0.132)	8.96 (0.117)	8.52 (0.127)
R_{int}/Ω cm^2		69.0 (0.359)	69.3 (0.419)	78.0 (0.425)	67.5 (0.357)
CPE_{int}	Y_o/10^{-7} Ω^{-1} cm^{-2} s^n	3.97 (0.195)	3.51 (0.200)	3.29 (0.163)	3.15 (0.166)
	n	0.980 (0.00434)	0.971 (0.00502)	0.979 (0.00438)	0.973 (0.00462)
R_e/Ω cm^2		167 (10.1)	47.4 (2.89)	76.1 (5.52)	71.3 (7.46)
CPE_e	Y_o/10^{-4} Ω^{-1} cm^{-2} s^n	1.77 (0.0899)	1.51 (0.199)	2.29 (0.223)	3.13 (0.303)
	n	0.930 (0.0154)	0.977 (0.0270)	0.917 (0.023)	0.904 (0.0262)
R_{ct}/Ω cm^2		9211 (856)	3088 (68.5)	1609 (30.1)	2835 (77.1)
CPE_{dl}	Y_o/10^{-4} Ω^{-1} cm^{-2} s^n	5.87 (0.0484)	4.94 (0.0416)	5.28 (0.0490)	5.21 (0.0426)
	n	0.805 (0.0122)	0.829 (0.00616)	0.856 (0.00920)	0.800 (0.00975)

Table S3. Electrochemical kinetic parameters for MPBI and 2.25Pt/MPBI in CH_2Cl_2/0.1 M $TBAPF_6$ at room temperature.

**Parameters**	**MPBI**		**2.25Pt/MPBI**	
	**Oxidation**	**Reduction**	**Oxidation**	**Reduction**
E_p (V *vs*. Ag/Ag$^+$, ν = 100 mV s^{-1})	0.81	0.15	1.02	0.55
α	0.053	0.083	0.32	0.22
D (cm^2 s^{-1})	0.021	0.044	0.33	0.72
k^0 (cm s^{-1})	0.11	0.30	0.82	0.93

Supplementary References

[S1] S. Park, W.J. Chang, C.W. Lee, S. Park, H.-Y. Ahn, and K.T. Nam, "Photocatalytic hydrogen generation from hydriodic acid using methylammonium lead iodide in dynamic equilibrium with aqueous solution," *Nature Energy* 2, no. 1 (2017): 16185. https://doi.org/10.1038/nenergy.2016.185

[S2] X. Wang, H. Wang, H. Zhang, et al., "Dynamic interaction between methylammonium lead iodide and TiO_2 nanocrystals leads to enhanced photocatalytic H_2 evolution from HI splitting," *ACS Energy Letters* 3, no. 5 (2018): 1159-1164. https://doi.org/10.1021/acsenergylett.8b00488

[S3] Y. Wu, P. Wang, Z. Guan, et al., "Enhancing the photocatalytic hydrogen evolution activity of mixed-halide perovskite $CH_3NH_3PbBr_{3-x}I_x$ achieved by bandgap funneling of charge carriers," *ACS Catalysis* 8, no. 11 (2018): 10349-10357. https://doi.org/10.1021/acscatal.8b02374

[S4] Y. Guo, G. Liu, Z. Li, Y. Lou, J. Chen, and Y. Zhao, "Stable lead-free $(CH_3NH_3)_3Bi_2I_9$ perovskite for photocatalytic hydrogen generation," *ACS Sustainable Chemistry & Engineering* 7, no. 17 (2019): 15080-15085. https://doi.org/10.1021/acssuschemeng.9b03761

[S5] G. Chen, P. Wang, Y. Wu, et al., "Lead-free halide perovskite $Cs_3Bi_{2x}Sb_{2-2x}I_9$ ($x \approx 0.3$) possessing the photocatalytic activity for hydrogen evolution comparable to that of $(CH_3NH_3)PbI_3$," *Advanced Materials* 32, no. 39 (2020): 2001344. https://doi.org/10.1002/adma.202001344

[S6] P. Zhou, H. Chen, Y. Chao, et al., "Single-atom Pt−I_3 sites on all-inorganic Cs_2SnI_6 perovskite for efficient photocatalytic hydrogen production," *Nature Communications* 12, no. 1 (2021): 4412. https://doi.org/10.1038/s41467-021-24702-8

[S7] H. Zhao, K. Chordiya, P. Leukkunen, et al., "Dimethylammonium iodide stabilized bismuth halide perovskite photocatalyst for hydrogen evolution," *Nano Research* 14, no. 4 (2021): 1116-1125. https://doi.org/10.1007/s12274-020-3159-0

[S8] H. Wang, H. Zhang, J. Wang, et al., "Mechanistic understanding of efficient photocatalytic H_2 evolution on two-dimensional layered lead iodide hybrid perovskites," *Angewandte Chemie International Edition* 60, no. 13 (2021): 7376-7381. https://doi.org/10.1002/anie.202014623

[S9] Y. Wu, Q. Wu, Q. Zhang, et al., "An organometal halide perovskite supported Pt single-atom photocatalyst for H_2 evolution," *Energy & Environmental Science* 15, no. 3 (2022): 1271-1281. https://doi.org/10.1039/D1EE03679C

[S10] H. Fu, X. Liu, J. Fu, et al., "2D/quasi-2D ruddlesden–popper perovskite: A high-performance photocatalyst for hydrogen evolution," *ACS Catalysis* 13, no. 22 (2023): 14716-14724. https://doi.org/10.1021/acscatal.3c03933

[S11] S. Gao, B. Wang, F. Chen, et al., "Confinement of $CsPbBr_3$ perovskite nanocrystals into extra-large-pore zeolite for efficient and stable photocatalytic hydrogen evolution," *Angewandte Chemie International Edition* 63, no. 15 (2024): e202319996. https://doi.org/10.1002/anie.202319996

[S12] J. Chen, C. Sun, Y. Xiang, X.-L. Wang, and Y.-F. Yao, "Bismuth-doped methylamine lead bromide perovskite $CH_3NH_3PbBr_3$ single crystals for efficient hydrogen evolution via hydrobromic acid splitting," *Journal of Colloid and Interface Science* 693, no. (2025): 137567. https://doi.org/10.1016/j.jcis.2025.137567

[S13] X. Wang, X. Sui, M. Wang, et al., "Facet junction of two-dimensional metal halide perovskite for photocatalytic hydrogen evolution," *Applied Catalysis B: Environment and Energy* 365, no. (2025): 124937. https://doi.org/10.1016/j.apcatb.2024.124937

[S14] J.P. Perdew, K. Burke, and M. Ernzerhof, "Generalized gradient approximation made simple," *Physical Review Letters* 77, no. 18 (1996): 3865-3868. https://doi.org/10.1103/PhysRevLett.77.3865

[S15] P.E. Blöchl, "Projector augmented-wave method," *Physical Review B* 50, no. 24 (1994): 17953-17979. https://doi.org/10.1103/PhysRevB.50.17953

[S16] G. Kresse, and D. Joubert, "From ultrasoft pseudopotentials to the projector augmented-wave method," *Physical Review B* 59, no. 3 (1999): 1758-1775. https://doi.org/10.1103/PhysRevB.59.1758

[S17] G. Kresse, and J. Furthmüller, "Efficient iterative schemes for ab initio total-energy calculations using a plane-wave basis set," *Physical Review B* 54, no. 16 (1996): 11169-11186. https://doi.org/10.1103/PhysRevB.54.11169

[S18] G. Kresse, and J. Furthmüller, "Efficiency of ab-initio total energy calculations for metals and semiconductors using a plane-wave basis set," *Computational Materials Science* 6, no. 1 (1996): 15-50. https://doi.org/10.1016/0927-0256(96)00008-0

[S19] S. Grimme, J. Antony, S. Ehrlich, and H. Krieg, "A consistent and accurate ab initio parametrization of density functional dispersion correction (DFT-D) for the 94 elements H-Pu," *The Journal of Chemical Physics* 132, no. 15 (2010): 154104. https://doi.org/10.1063/1.3382344

[S20] J.K. Nørskov, J. Rossmeisl, A. Logadottir, et al., "Origin of the overpotential for oxygen reduction at a fuel-cell cathode," *The Journal of Physical Chemistry B* 108, no. 46 (2004): 17886-17892. https://doi.org/10.1021/jp047349j

[S21] K. Mathew, V.S.C. Kolluru, S. Mula, S.N. Steinmann, and R.G. Hennig, "Implicit self-consistent electrolyte model in plane-wave density-functional theory," *The Journal of Chemical Physics* 151, no. 23 (2019): 234101. https://doi.org/10.1063/1.5132354

[S22] G. Schuck, D.M. Többens, M. Koch-Müller, I. Efthimiopoulos, and S. Schorr, "Infrared spectroscopic study of vibrational modes

across the orthorhombic–tetragonal phase transition in methylammonium lead halide single crystals," *The Journal of Physical Chemistry C* 122, no. 10 (2018): 5227-5237. https://doi.org/10.1021/acs.jpcc.7b11499

[S23] D.M. Jang, K. Park, D.H. Kim, et al., "Reversible halide exchange reaction of organometal trihalide perovskite colloidal nanocrystals for full-range band gap tuning," *Nano Letters* 15, no. 8 (2015): 5191-5199. https://doi.org/10.1021/acs.nanolett.5b01430

[S24] A.R.C. Bredar, A.L. Chown, A.R. Burton, and B.H. Farnum, "Electrochemical impedance spectroscopy of metal oxide electrodes for energy applications," *ACS Applied Energy Materials* 3, no. 1 (2020): 66-98. https://doi.org/10.1021/acsaem.9b01965

[S25] Q.-C. Zhuang, T. Wei, L.-L. Du, Y.-L. Cui, L. Fang, and S.-G. Sun, "An electrochemical impedance spectroscopic study of the electronic and ionic transport properties of spinel $LiMn_2O_4$," *The Journal of Physical Chemistry C* 114, no. 18 (2010): 8614-8621. https://doi.org/10.1021/jp9109157

[S26] Y. Tang, C.H. Mak, J. Zhang, et al., "Unravelling the interfacial dynamics of bandgap funneling in bismuth-based halide perovskites," *Advanced Materials* 35, no. 2 (2023): 2207835. https://doi.org/10.1002/adma.202207835

[S27] H. Huang, B. Pradhan, J. Hofkens, M.B.J. Roeffaers, and J.A. Steele, "Solar-driven metal halide perovskite photocatalysis: Design, stability, and performance," *ACS Energy Letters* 5, no. 4 (2020): 1107-1123. https://doi.org/10.1021/acsenergylett.0c00058

[S28] X. Zhao, S. Chen, H. Yin, et al., "Perovskite microcrystals with intercalated monolayer MoS_2 nanosheets as advanced photocatalyst for solar-powered hydrogen generation," *Matter* 3, no. 3 (2020): 935-949. https://doi.org/10.1016/j.matt.2020.07.004
